# Identifying intracellular signaling modules and exploring pathways associated with breast cancer recurrence

**DOI:** 10.1038/s41598-020-79603-5

**Published:** 2021-01-11

**Authors:** Xi Chen, Jinghua Gu, Andrew F. Neuwald, Leena Hilakivi-Clarke, Robert Clarke, Jianhua Xuan

**Affiliations:** 1 Bradley Department of Electrical and Computer Engineering, Virginia Polytechnic Institute and State University, 900 North Glebe Road, Arlington, VA 22203 USA; 2 Center for Computational Biology, Flatiron Institute, Simons Foundation, 162 Fifth Avenue, New York, NY 10010 USA; 3 Institute for Genome Sciences and Department Biochemistry and Molecular Biology, University of Maryland School of Medicine, 670 W. Baltimore Street, Baltimore, MD 21201 USA; 4 Hormel Institute, University of Minnesota, 801 16th Ave NE, Austin, MN 55912 USA

**Keywords:** Computational biology and bioinformatics, Cellular signalling networks, Statistical methods

## Abstract

Exploring complex modularization of intracellular signal transduction pathways is critical to understanding aberrant cellular responses during disease development and drug treatment. IMPALA (Inferred Modularization of PAthway LAndscapes) integrates information from high throughput gene expression experiments and genome-scale knowledge databases to identify aberrant pathway modules, thereby providing a powerful sampling strategy to reconstruct and explore pathway landscapes. Here IMPALA identifies pathway modules associated with breast cancer recurrence and Tamoxifen resistance. Focusing on estrogen-receptor (ER) signaling, IMPALA identifies alternative pathways from gene expression data of Tamoxifen treated ER positive breast cancer patient samples. These pathways were often interconnected through cytoplasmic genes such as IRS1/2, JAK1, YWHAZ, CSNK2A1, MAPK1 and HSP90AA1 and significantly enriched with ErbB, MAPK, and JAK-STAT signaling components. Characterization of the pathway landscape revealed key modules associated with ER signaling and with cell cycle and apoptosis signaling. We validated IMPALA-identified pathway modules using data from four different breast cancer cell lines including sensitive and resistant models to Tamoxifen. Results showed that a majority of genes in cell cycle/apoptosis modules that were up-regulated in breast cancer patients with short survivals (< 5 years) were also over-expressed in drug resistant cell lines, whereas the transcription factors JUN, FOS, and STAT3 were down-regulated in both patient and drug resistant cell lines. Hence, IMPALA identified pathways were associated with Tamoxifen resistance and an increased risk of breast cancer recurrence. The IMPALA package is available at https://dlrl.ece.vt.edu/software/.

## Introduction

A new direction^1,2^ in the design of anti-cancer drug therapies is to "globally" target multiple genes involved in crosstalk among various cancer-associated signaling pathways^3^ rather than the traditional approach of targeting a single molecular pathway. For example, BIRC5 intersects multiple pathways essential for cell proliferation, survival, and resistance to growth inhibition^3^. The goal is to identify anticancer drugs that interfere with multiple molecular targets in different subcellular compartments while minimizing damage to normal cells^1,4,5^. However, to be effective, such combinatorial drug design must address the complexity and heterogeneity inherent in most cancers, which, in turn, requires the development of systems biology tools to characterize multiple cancer-specific pathways and signaling networks^6^. Although there are computational methods for deciphering complex signal transduction pathways by integrating multi-platform genomic data with biological knowledge like GESA^7^ and PARADIGM^8^, their ability to discover novel pathway interactions is limited.

The current abundance of genome-wide protein–protein interaction (PPIs) data^9^ provides an alternative source of information for signaling pathway identification, which typically has been formulated as a mathematical problem of reconstructing paths between source and target genes^10^. The main challenge for such methods—which include, for example, Netsearch^11^, random color coding^12^, integer linear programming (ILP)^10^ and ResponseNet^13,14^—is inferring signaling directions between genes given non-directed PPI network information. Gitter et al. proposed to use maximum edge orientation (EO) on a PPI network to determine the most likely signaling directions that fulfil global optimality^15^. However, EO relies heavily on the assumption that most biological pathways are short (length < 5) in order to accommodate the requirement of exhaustive enumeration of possible pathways and fails to utilize important biological knowledge such as subcellular information. Hence, assigned signaling directions are usually difficult to interpret in a biological meaningful way. Furthermore, EO fails to jointly analyze individual pathways for structural or functional similarities, which are important for studying pathway crosstalk.

IMPALA (Inferring Modularization of PAthway LAndscape) integrates gene expression data and biological knowledge within a Bayesian framework to reconstruct aberrant pathway modules. IMPALA defines three potential functions representing gene expression, gene co-expression and prior network interactions. These functions, which jointly measure the aberrancy of individual pathways, are converted to probability distributions for pathway sampling. IMPALA estimates edge directions by aggregating pathway samples. To study crosstalk between multiple pathways, sampled pathways are clustered into interconnected modules based on structural similarities.

Here we use IMPALA to identify and explore estrogen-receptor (ER) signaling associated with Tamoxifen resistance in breast cancer and to build an aberrant pathway network connecting ER to transcription factors involved in cell proliferation and apoptosis. The identified pathway network was significantly enriched in ErbB, MAPK and JAK-STAT signaling components. Pathway clustering by IMPALA identified key functionally associated ER signaling, cell cycle and apoptosis modules with crosstalk. We validated the expression of module genes using breast cancer cell line models. Hence, IMPALA provides a novel and effective approach to investigate alternative pathways and pathway crosstalk in cancer cells.

## Results

### Identifying aberrant signaling pathway transduction in Tamoxifen-treated breast cancer patients

**Figure 1:**
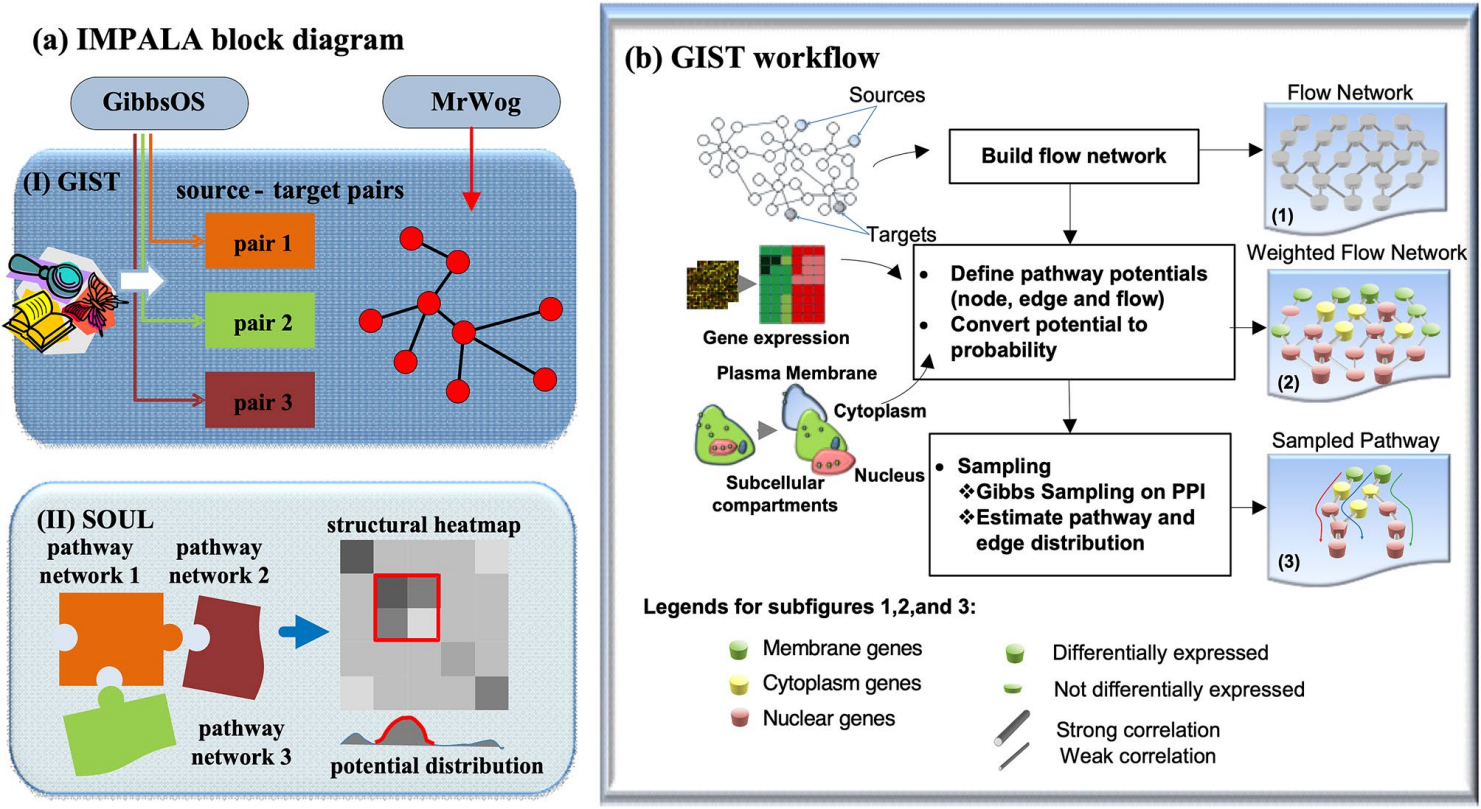
IMPALA block diagram and GIST workflow. (**a**) Key transcription factors and the candidate pathway landscape are identified using GibbsOS and MrWOG to pre-process gene expression and protein–protein interaction data (HPRD database). Then, IMPALA integrates gene expression and candidate pathways to identify aberrant signal pathway transduction using GIST (Gibbs sampler to Infer Signal Transduction) and pathway modules using SOUL (Structural Organization to Uncover pathway Landscape). (**b**) GIST integrates gene (node), gene–gene interaction (edge) and network flow potentials to build a weighted and directed Bayesian network and infers signaling directions between genes using Gibbs Sampling.

IMPALA is a Bayesian approach to infer signaling pathway modules from gene expression data (Fig. 1). We applied IMPALA to a gene expression (microarray) dataset (termed Loi) including samples from Tamoxifen-treated ER positive breast cancer patients^16^ and identified aberrant signal pathway transduction associated with Tamoxifen resistance. We normalized the data using PLIER (http://www.affymetrix.com), and then corrected the batch effects using ComBat^17^. A 5-year cut-off on distant-metastasis-free-survival (DMFS) was used to divide Loi samples into ‘early recurrence’ (DMFS ≤ 5 years) and ‘late recurrence’ (DMFS > 5 years) groups, yielding 88 and 92 samples, respectively.

IMPALA utilizes two functional components: (1) Gibbs sampling to Infer Signal Transduction (GIST) and (2) Structural Organization to Uncover pathway Landscape (SOUL) (Fig. 1a). GIST reconstructs pathways (genes and directed interactions) related to ER signaling. Specifically, using MrWOG^18^ a gene network was extracted from protein–protein interaction data to predict genes and interactions likely associated with ER signaling. Candidate pathways were constructed starting from the estrogen receptor ESR1 gene and targeting breast cancer-associated transcription factors, such as JUN, FOS, STAT1, STAT3, STAT5A, ELK1, and ETS1 (Target transcription factors were pre-identified by GibbsOS^19^; see Supplementary Tables S1 and S2). GIST uses a Bayesian framework to integrate candidate pathways with gene expression data and uses Gibbs sampling to iteratively infer signaling pathways (Fig. 1b).

**Figure 2:**
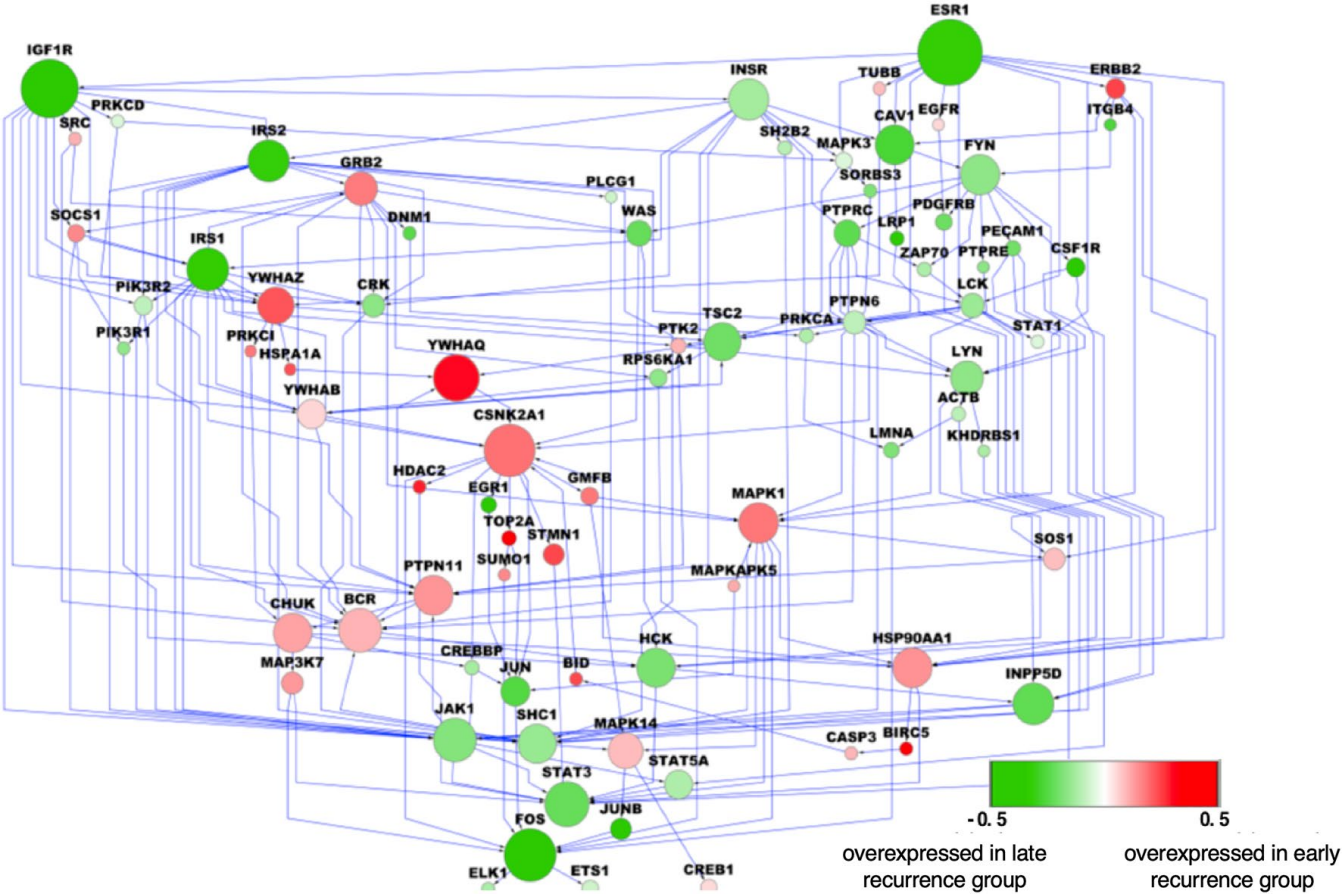
An ER signaling pathway network identified by IMPALA using Loi breast cancer gene expression data. The gene color represents the log_2_(*x*)-fold change of gene expression between early and late recurrence groups of patients in the Loi dataset (red: over-expressed in ‘early recurrence’ group; green: over-expressed in ‘late recurrence’ group). Gene’s size is proportional to the probability (sampling frequency) estimated by GIST.

A directed pathway network assembled by collapsing the top 200 GIST pathway samples is shown in Fig. 2. This reveals complex wiring of alternative pathways that are interconnected through frequently sampled cytoplasmic genes, such as IRS1/2, JAK1, YWHAZ, CSNK2A1, MAPK1 and HSP90AA1. Functional enrichment analysis using DAVID^20^ returned, as significant, canonical insulin (*p*-value 2.4e−10), ErbB (*p*-value 4.0e−13), MAPK (*p*-value 5.1e−8), and JAK-STAT (*p*-value 2.0e−5) signaling pathways, each of which plays a key role in breast cancer^21^. We further examined the association of the pathway network with Tamoxifen recurrence by using the network to predict the survival of breast cancer patients based on a similar, but independently generated gene expression dataset (termed Symmans)^22^. Specifically, using the above ER signaling pathway network and the Loi gene expression data, we trained a NetSVM classifier^23^ to group samples as early or late. Threefold cross-validation using Loi data returned the area under ROC curve (AUC) as 0.8. Applying the classifier to the Symmans dataset, which includes 103 patient samples, we obtained a prediction AUC of 0.79. Kaplan Meier analysis of Symmans data returned a hazard ratio of 3.26 (*p*-value = 0.016; Supplementary Fig. S2).

### Identifying pathway modules and crosstalk

**Figure 3:**
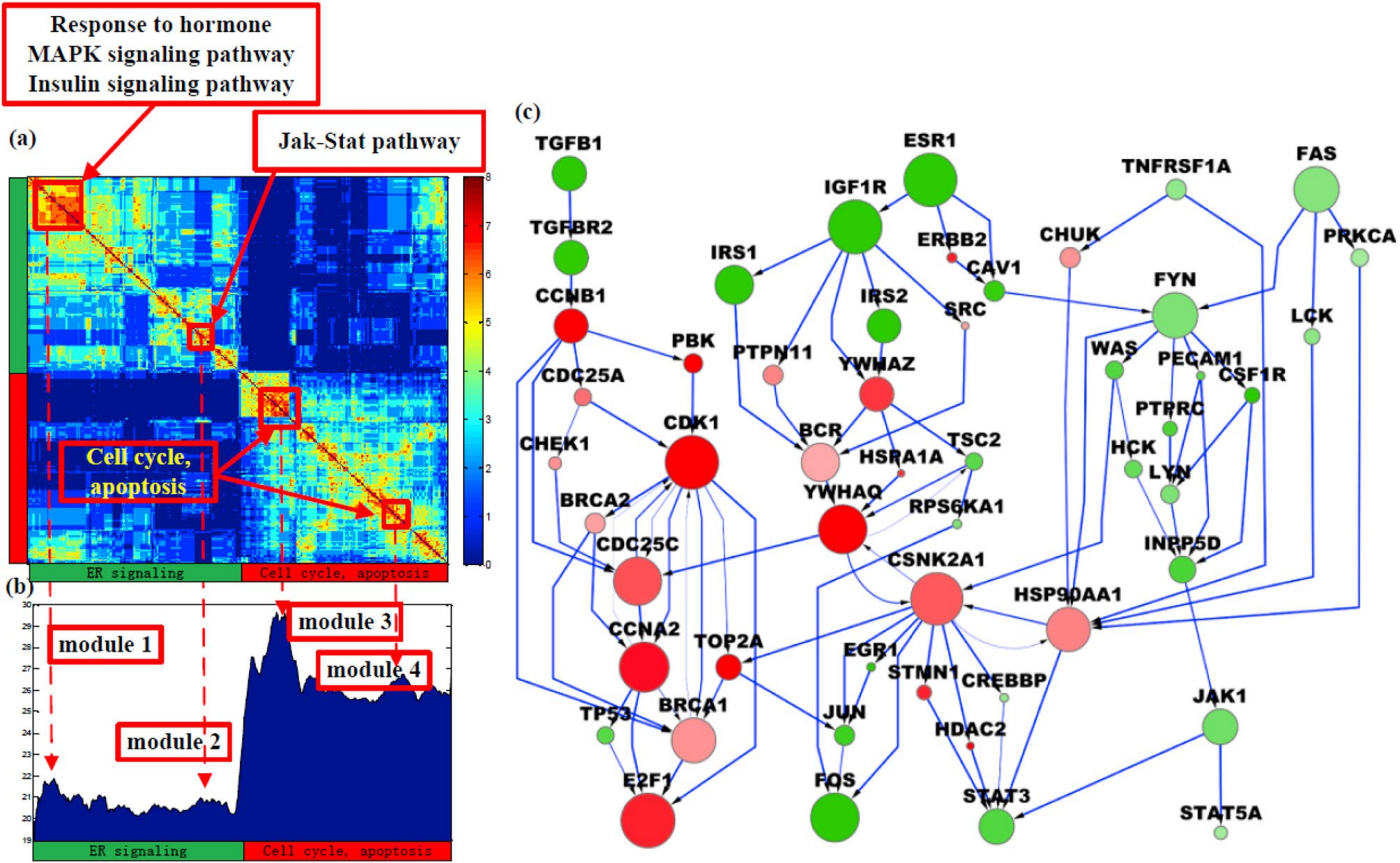
Pathway modules and crosstalk identified by IMPALA for the Loi dataset. (**a**) Pathway clustering based on gene similarity and gene functions in different clusters reveal the functional diversity of IMPALA-identified pathways. (**b**) Distribution of sampling frequency of pathways with peaks corresponding to major pathway clusters in (**a**). Four pathway modules were identified. (**c**) A combined pathway network consisting of the four modules with crosstalk.

To study crosstalk between ER signaling and cancer cell proliferation, we further used GIST to identify cell cycle and apoptosis signaling modules (Supplementary Fig. S1). We used the SOUL component of IMPALA to analyze pooled samples from GIST and to investigate and assess the statistical significance of modules and crosstalk associated with ER, cell cycle, apoptosis signaling pathways, as shown in Fig. 3. SOUL hierarchically clustered sampled pathways based on gene overlap (Fig. 3a) and re-ordered the distribution of sampling frequency to be consistent with pathway clusters (Fig. 3b). Signaling modules were identified for each of four local peaks (modes) of the sample distribution, including two ER signaling modules (M1 and M2), one cell cycle module (M3) and one apoptosis module (M4). The specific genes in each module are listed in Supplementary Table S3. A pathway network of the four modules is shown in Fig. 3c. M1 is enriched with genes in response to hormones and also enriched with canonical MAPK and insulin signaling pathways. M2 corresponds to JAK-STAT signaling. The crosstalk between M3 and M4 is strong, as indicated by the pathway sample distribution. Although M4 contains genes functioning in apoptosis and cell death, it is also enriched with cell cycle genes, which suggests coupling of these cellular processes.

Genes upregulated in ‘early recurrence’ samples (survival ≤ 5 years) include signal transduction genes like YWHAQ, YWHAZ and PTPN11, the chaperone HSP90AA1, and STMN1, which functions in cytoskeletal rearrangements. HSP90AA1 is an intracellular gene that is actively expressed in breast cancer cells—high levels of which correlate with a low chance of survival^24^. Efficient progression through the cell cycle requires HSP90AA1^25^; when up-regulated in osteosarcoma it increases drug resistance by inducing autophagy and inhibiting apoptosis^26^. BRCA1 is a client gene of HSP90AA1, inhibition of which by 17-AAG Tanespimycin leads to degradation of BRCA1 via the ubiquitin–proteasome pathway. Subsequent loss of BRCA1 disrupts G2/M cell cycle checkpoint activation, resulting in mitotic catastrophe—an apoptosis-independent form of cell death caused by mechanical damage^27^. Thus, HSP90AA1 inhibition may promote survival in Tamoxifen-resistant tumors. STMN1 promotes catastrophes that ultimately lead to deregulation of the cell cycle, thereby hampering cell survival^28^. High STMN1 expression leads to shorter post-progression and overall survival in breast cancer patients^29^, consistent with our finding that STMN1 is up-regulated among tumor samples in the ‘early recurrence’ group (labelled ‘red’ in Fig. 3c). CDK1 is an essential modulator of the initiation of and progression through mitosis, acting primarily through its interaction with CCNB1. CDK1 and CCNB1 help protect mitotic cells against extrinsic death stimuli^30^. Thus, increased expression of CDK1 in early recurrence breast cancer may explain Tamoxifen resistance by protecting tumor cells from antiestrogen-mediated cell death.

We found ESR1 and IGF1R to be overexpressed in the ‘late recurrence’ group (‘green’ hub genes in Fig. 3c). Crosstalk between the IGF and ER signaling pathways is well known^31^. TSC2 is a negative regulator of mTOR, which in turn inhibits autophagy. Although cellular stress from therapeutic drugs can induce cell death via autophagy, lysosomal degradation or prolonged stress^32^ can sustain long-term survival or dormancy by enabling autophagy of some tumor cells^33^.

### Validating pathways and modules using Symmans breast cancer gene expression data

**Figure 4:**
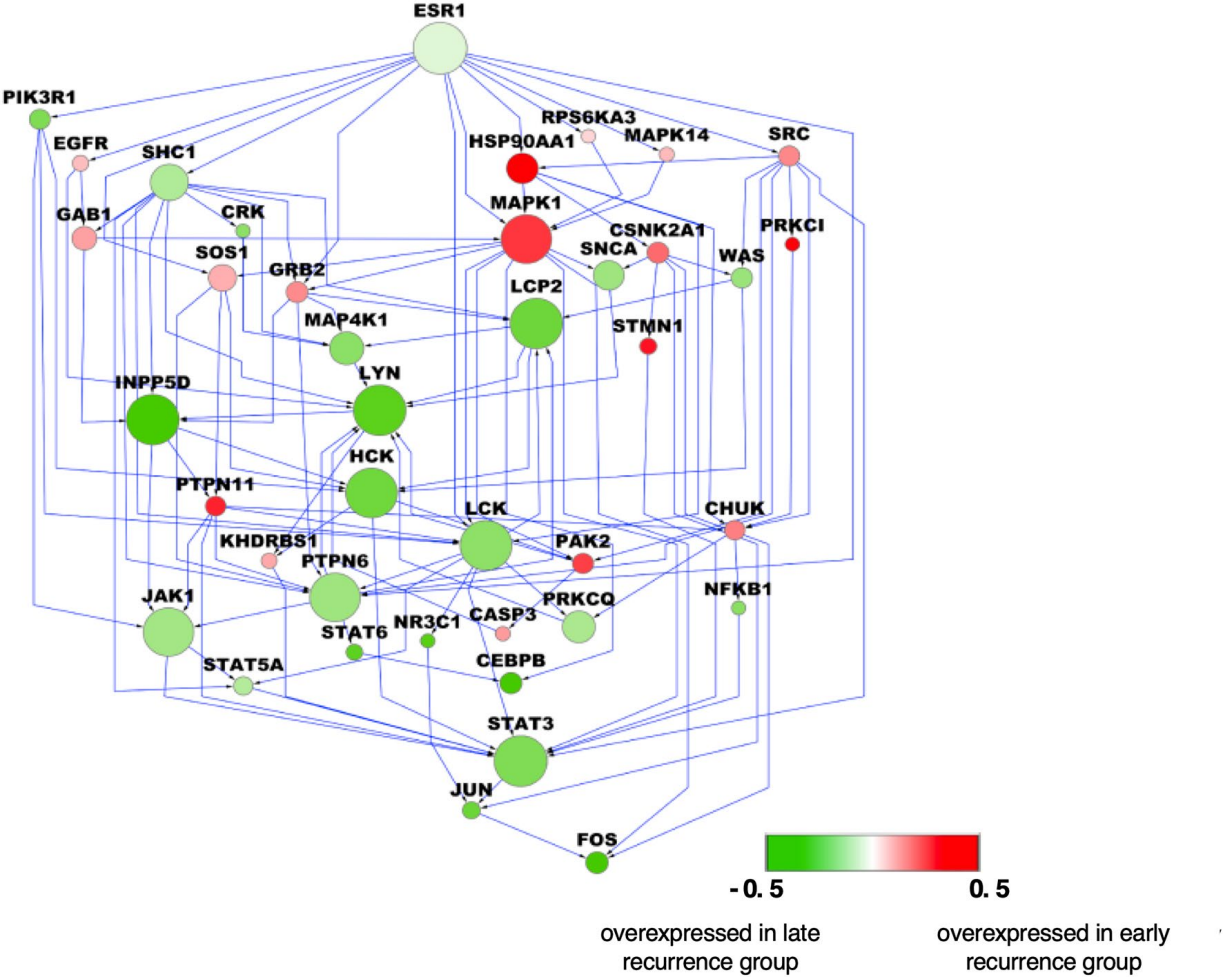
An ER signaling pathway network identified by IMPALA using Symmans data. Gene colors represent the log2 fold change of gene expression between ‘early recurrence’ and ‘late recurrence’ patients in the Symmans dataset (red: over-expressed in ‘early recurrence’ group; green: over-expressed in ‘late recurrence’ group). Gene size is proportional to the probability (sampling frequency) estimated by GIST.

To validate the robustness of IMPALA for characterizing networks associated with Tamoxifen resistance in breast cancer, we applied it to the Symmans dataset^22^ (Tamoxifen treated breast cancer gene expression (microarray) dataset; consisting of 47 ‘early recurrence’ and 56 ‘late recurrence’ samples based on a 5-year DMFS cutoff). Source receptor genes were the same as selected for the Loi data analysis, while target transcription factors were identified using GibbsOS for ER signaling, cell cycle, and apoptosis (Supplementary Table S4). Pathway networks of the top GIST-sampled pathways for ER signaling and for cell cycle and apoptosis are shown in Fig. 4 and in Supplementary Fig. S3, respectively. The similarity to genes in the Loi-based pathway networks for ER, cell cycle, and apoptosis signaling were 73%, 53% and 54%, respectively.

**Figure 5:**
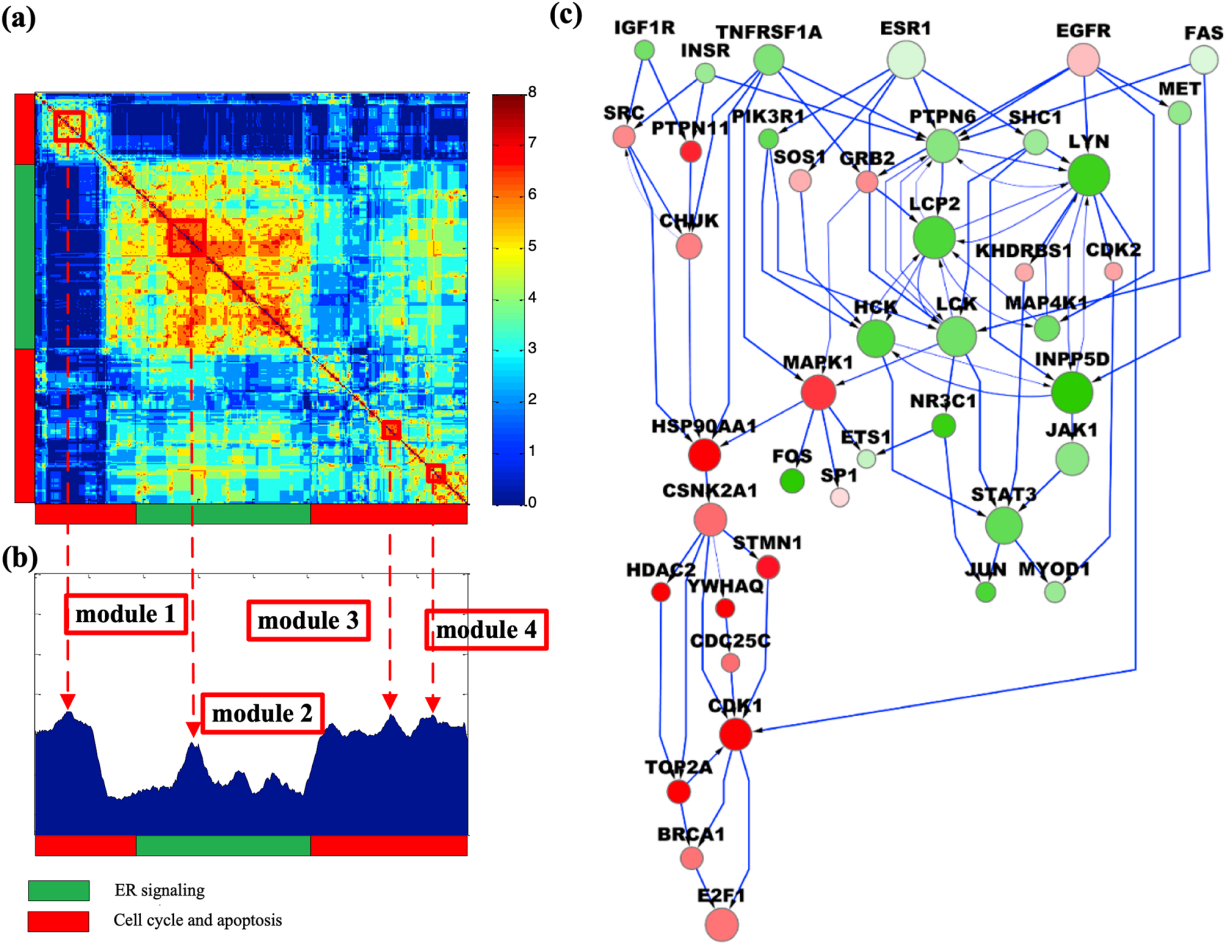
Pathway modules and crosstalk identified by IMPALA using the Symmans data. (**a**) Pathway clustering based on gene similarity and gene functions in different clusters reveal the functional diversity of IMPALA-identified pathways. (**b**) Distribution of sampling frequency of pathways with peaks corresponding to major pathway clusters in (a). Four pathway modules were identified. (**c**) A combined pathway network consisting of the four modules with crosstalk.

SOUL identified the four pathway modules (M1-M4) shown in Fig. 5. Specific genes in each module are listed in Supplementary Table S5. Again, we observed signal transductions from the membrane through cytoplasmic genes MAPK1, HSP90AA1, and CSNK2A1 to the nuclear transcription factors. In M1, signal pathways started from IGFR1 and INSR, passed through cytoplasmic signaling hubs SRC, CHUK, and HSP90AA1, and converged to the same targets within the nucleus. In M2 and M4, signal transduction took diverse pathways between membrane receptors and JAK-STAT activation. Signaling could be initiated by ESR1 via canonical members of the JAK-STAT pathway (PIK3R1, SOS1, and PTPN6), by various membrane receptors (INSR, EGFR), or by death receptors (FAS, TNFRSF1A) through PTPN6, SHC1, or LYN. Although M3 genes are mostly shared with M2 and M4, they form an alternative pathway for cell cycle progression genes (CDC2 and E2F1). Based on IMPALA pathway analyses of both the Loi and Symmans datasets, we conclude that HSP90AA1, CSNK2A1, and MAPK1 play key topological roles in intracellular signal transduction initiated by plasma membrane genes or canonical death receptors to regulate the cell cycle and apoptosis.

### Validating pathway gene expression in breast cancer cell line models

**Figure 6:**
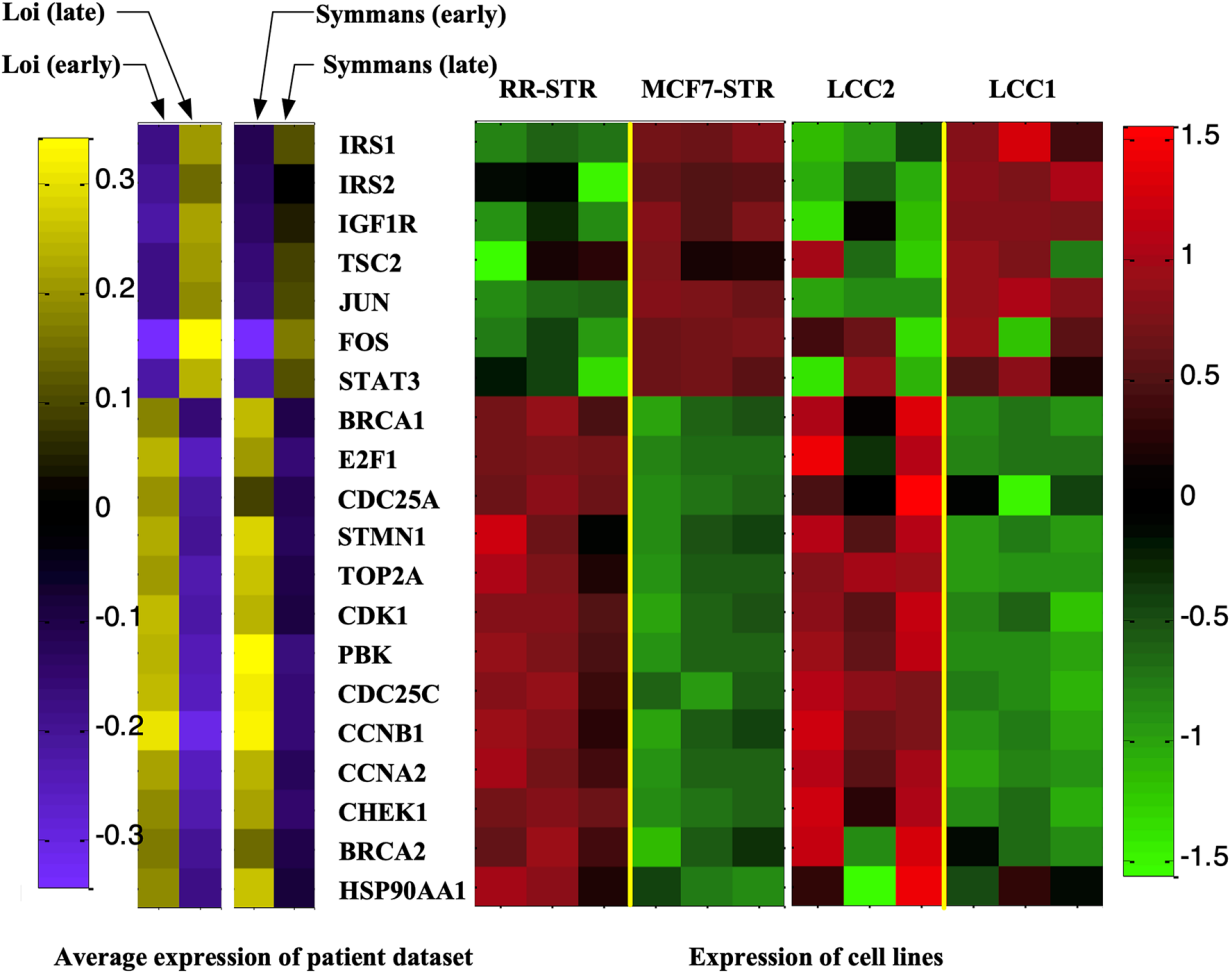
Cell line validation for identified pathway genes from patient datasets. The left panel shows the average log_2_ expression of selected pathway genes. The right panel shows the log_2_ expression of two cell line studies: (MCF7-STRP vs*.* MCF7RR-STRP and LCC1 vs. LCC2). Seven genes (IRS1, IRS2, IGF1R, TSC2, JUN, FOS, STAT3) are consistently over-expressed in the ‘early recurrence’ patient samples and sensitive human breast cancer cell lines. The remaining genes, which mainly relate to cell cycle and apoptosis, are over-expressed in the resistant groups.

We used in vitro breast cancer cell line models to validate the expression of genes in aberrant pathway modules identified by IMPALA. Four MCF7 derived cell models were included in the analysis: MCF7-STR, MCF7RR-STR, LCC1, and LCC2^34^. MCF7RR-STR and LCC2 are Tamoxifen resistant, whereas MCF7-STR and LCC1 are sensitive. As shown in Fig. 6, 20 genes from IMPALA-identified pathway modules exhibited consistent expression patterns between patient data and cell line data. ER signaling genes, such as STMN1, PBK, CCNB1 and HSP90AA1, were overexpressed in early recurrence/resistant groups, whereas IRS1, IRS2, IGF1R and TSC2 were overexpressed in the ‘late recurrence/drug-sensitive’ groups. The cell cycle/apoptosis genes BRCA1, BRCA2, CCNA2, E2F1, CDC25A, CDC25C, TOP2A, CDC2, and CHUK were up-regulated in the ‘early recurrence’ group and also in the Tamoxifen resistant cell lines, whereas the transcription factors JUN, FOS, and STAT3 were down-regulated. Gene expression for in vitro cell lines identified from Loi and Symmans datasets are shown in Supplementary Figures S4 and S5, respectively. The concordance between patient and cell line data demonstrates the association of IMPALA identified pathways with Tamoxifen resistance and with increased breast cancer recurrence.

## Discussion

IMPALA characterizes intracellular signal transduction pathways by integrating multi-platform data and by identifying crosstalk among pathways. Using this approach, we identified breast cancer-associated aberrant pathways by integrating breast cancer gene expression data with protein-DNA and protein–protein interaction data, and with published information regarding signaling pathways.

IMPALA has several notable advantages over existing methods. First, GIST allows users to incorporate the subcellular location of genes in order to focus on signal transduction components in the nucleus, the cytoplasm, or the plasma membrane. Second, most existing methods either fail to assign signaling directions between genes or else infer signaling direction in an ad hoc manner. GIST assigns a posterior probability for each signaling direction, thereby estimating a degree of confidence. Third, SOUL models network components as structurally related modules to better identify local modules within a large-scale pathway landscape. This identifies overlap between modules, which corresponds to crosstalk between pathways.

Unravelling signaling pathways from complex molecular networks in cancer cells is challenging^35^. Here, IMPALA revealed that breast cancer-associated pathway modules are structurally interconnected with crosstalk between ER signaling, cell cycle and apoptosis pathways, thereby imparting tamoxifen resistance. And, by characterizing the pathway landscape, IMPALA systematically categorized complex pathway interactions into within-module and between-module interactions. This echoes the increasing emphasis among researchers on networks, rather than pathways, as a reflection of the complex and integrated nature of molecular signaling.

## Methods

IMPALA applies GIST to identify signaling pathways by integrating gene expression data with protein–protein interactions (PPIs), and SOUL to explore the pathway landscape for pathway module and crosstalk identification.

### Identifying source and target genes for pathway exploration

To build the candidate pathway landscape, we pre-selected the source and target genes for each signaling pathway. Specifically, we selected ESR1 for ER signaling, membrane receptors and the growth factors EGFR, TGFB1, IGF1R, INSR, FGFR1 for cell cycle, and canonical death receptors IL1R1, FAS, and TNFRSF1A for apoptosis. Based on literatures, we selected transcription factors associating to breast cancer recurrence as pathway targets. Categorized transcription factors selected for ER signaling, cell cycle, and apoptosis are listed in Supplementary Table S1. To refine the candidate target genes, we applied GibbsOS^36^ to the Loi and Symmans datasets, respectively, and selected transcription factors significantly associated with the survival difference, as listed in Supplementary Tables S2 and S4.

### Building the candidate pathway landscape using MRWOG

To build a candidate pathway landscape, we used MRWOG^18^ to pre-screen human PPIs for an ER-related, Tamoxifen resistant sub-network. An ESR1-centered PPI subnetwork including 2326 genes (all genes within a two-step distance from ESR1) was selected.

### The GIST algorithm

To infer signal directions between genes, GIST constructs a flow network of a given pathway length between source and target genes. To weight the flow network, node (gene), edge (interaction) and flow (network) potentials are defined for individual pathways. GIST converts the three potentials into a joint probability distribution so that samples of candidate pathways can be drawn probabilistically. Signaling pathway directions were inferred by aggregating the pathways samples and then selecting the interconnected linear pathways with the largest potentials.

We define a vector ${{\varvec{\uptheta}}}_{1 \times L} = \left\{ {\theta_{1} ,\;\theta_{2} , \ldots ,\;\theta_{L} } \right\}$ to represent a linear pathway with length *L* genes, where $\theta_{i}$ is a categorical variable representing the *i*th gene in the pathway. $\theta_{1}$ and $\theta_{L}$ are the source and target genes, respectively. Let $\Omega_{i}$ denote the domain of $\theta_{i}$ and we have $\Omega_{1} \bigcap {\Omega_{2} } \bigcap { \cdots \bigcap {\Omega_{L} } } \subseteq \Omega$, where the full domain $\Omega$ denotes the whole set of genes in the PPI dataset. Given gene expression data ${\mathbf{X}}_{n \times m}$, which includes *n* genes and *m* samples with two conditions (to study aberrant signal pathway transduction between conditions), we derive gene potential ${\text{V}}_{1} (\theta_{i} ;\;{\mathbf{X}})$, defined as the sum of pathway gene differential expression z-scores between the two types^37^; edge potential ${\text{V}}_{2} (\theta_{i} ,\;\theta_{i + 1} ;\;{\mathbf{X}})$, defined as the sum of z-scores calculated from the statistical significance of Pearson’s correlation between interacting genes^38^; and flow potential ${\text{V}}_{3} ({{\varvec{\uptheta}}})$, defined as a proportionally score reflecting the concordance between a pathway and prior information regarding cellular location^39^. Derivations of the three potentials are provided in the Supplementary Methods.

GIST integrates the three potentials into a pathway energy function as follows:

$${\text{U}} ({{\varvec{\uptheta}}};\;{\mathbf{X}}) = \sum\limits_{i = 1}^{L} {{\text{V}}_{1} (\theta_{i} ;\;{\mathbf{X}})} + \sum\limits_{i = 1}^{L - 1} {{\text{V}}_{2} (\theta_{i} ,\;\theta_{i + 1} ;\;{\mathbf{X}})} + {\text{V}}_{3} ({{\varvec{\uptheta}}}).$$

Due to the large number of genes and their interactions, finding the optimal solution of Eq. (1) is a NP hard problem. Therefore, we convert the optimization task into a distribution learning problem as show in Eq. (2) and used Gibbs sampling to search for the optimal solution.

2 $$\begin{aligned} P({{\varvec{\uptheta}}};\;{\mathbf{X}}) & = \frac{1}{Z} \cdot e^{{\frac{{ - {\text{S}} ({{\varvec{\uptheta}}};\;{\mathbf{X}})}}{T}}} = \frac{1}{Z} \cdot e^{{\frac{{{\text{U}} ({{\varvec{\uptheta}}};\;{\mathbf{X}})}}{T}}} \\ & = \frac{1}{Z} \cdot \exp \left( {\frac{{\sum\limits_{i = 1}^{L} {{\text{V}}_{1} (\theta_{i} ;\;{\mathbf{X}})} + \sum\limits_{i = 1}^{L - 1} {{\text{V}}_{2} (\theta_{i} ,\;\theta_{i + 1} ;\;{\mathbf{X}})} + {\text{V}}_{3} ({{\varvec{\uptheta}}})}}{T}} \right), \\ \end{aligned}$$

where $Z = \sum\nolimits_{{{{\varvec{\uptheta}}} \in {{\varvec{\Theta}}}}} {e^{{\frac{1}{T}{\text{U}} \left( {{{\varvec{\uptheta}}};{\mathbf{X}}} \right)}} }$ is a partition function and *T* is the "temperature" that controls the shape of the distribution. GIST samples pathway genes iteratively from a conditional distribution as $\theta_{i}^{(t + 1)} \sim P(\left. {\theta_{i} } \right|\theta_{1}^{(t + 1)} , \ldots ,\theta_{i - 1}^{(t + 1)} ,\;\theta_{i + 1}^{(t)} ,\; \ldots \theta_{L}^{(t)} ;\;{\mathbf{X}})$. In each iteration, it probabilistically samples $\theta_{i}$ conditioned on the other, currently assigned genes $\theta_{ - i}$ in the pathway. After the sampler appears to have converged to a stationary distribution, GIST accumulates samples from this conditional distribution to approximate the posterior distribution. Details about GIST sampling are provided in Supplementary Methods, Figures S7 and S8.

After 10,000 iterations, GIST pools the pathway samples and then estimates edge directions. We introduce a binary variable $e_{i,j}$ to denote the signaling direction from gene $\omega_{i} \in \Omega$ to gene $\omega_{j} \in \Omega$. The probability of $e_{i,j}$ is estimated as follows:

3 $$p_{i,j}^{*} = P(e_{i,j} = 1) = \sum\limits_{{{{\varvec{\uptheta}}} \in {{\varvec{\Theta}}}}}^{{}} {P(e_{i,j} = 1\left| {{\varvec{\uptheta}}} \right.)P({{\varvec{\uptheta}}})} ,$$

where $P(e_{i,j} = 1\left| {{\varvec{\uptheta}}} \right.) = 1$ if $e_{i,j}$ corresponds to a connected edge in pathway ${{\varvec{\uptheta}}}$; otherwise it equals 0. Using Eq. (3), GIST models each directed edge as a Bernoulli random variable with success rate $p_{i,j}$ . It performs both forward and reverse searching so that the probabilities of edge direction from gene *i* to gene *j* and its reverse direction are both estimated (Supplementary Methods, Fig. S6). If $p_{i,j}$ is close to 1, the signal flows from gene $\omega_{i}$ to gene $\omega_{j}$ with high confidence, while $p_{i,j}$ = 0.5 indicates a lack of confidence in the direction of signal flow.

### The SOUL algorithm

SOUL post-processes distributions of GIST pathway samples to reconstruct the overall landscape. Given thousands of genes, the pathway sample distribution can be multi-modal and some hub genes (i.e., those involved in multiple pathways more often than others) could bias the sample distribution. Instead of directly ranking pathways based on their GIST sampling frequency, SOUL first clusters pathway samples based on their structural similarities using hierarchical clustering, resulting in a re-organized pathway topological pattern visualized as a pathway structural heatmap (as in Fig. 3a). Next, SOUL re-orders the pathway sampling frequencies to be consistent with pathway clusters (as in Fig. 3b). Finally, it identifies high-confidence pathway modules from local peaks in the pathway sampling frequency distribution.

### IMPALA performance evaluation on simulated data

We evaluated the performance of GIST for pathway identification on simulated datasets generated by two different pathway structures: type I, corresponding to alternative pathways between a single source gene and a single target gene; and type II, corresponding to multiple pathways with crosstalk among multiple sources and targets (Supplementary Fig. S9). PPI data from the HPRD database^40,41^ and canonical pathways from the KEGG database^42^ were used to simulate pathways that include 261 genes and 998 interactions for type I, and 266 genes and 1026 interactions for type II. We added noise to gene expression data (Gaussian distributed noise with zero-mean and variance varying from 0.2 to 0.8, compared to the gene expression data) and to simulated pathway networks (false gene interactions varying from 10 to 50%, compared to the ‘true’ interactions).

Supplementary Figures S10–S13 and Tables S6 and S7 summarize the performance of IMPALA versus three competing algorithms: random color coding^12^, edge orientation^15^, and integer linear programming (ILP)^10^. Note that we only applied ILP to pathway gene identification because ILP does not infer signaling directions. IMPALA consistently obtained comparable or better performance in all cases. When the level of noise was set to 0.2 (20% false interactions in the network), IMPALA gained about a 16% increase in precision for type I pathway gene identification, and an even larger improvement of 24% for edge identification. Similarly, for type II GIST achieved about a 15% increase in average precision for gene identification, and a 17% increase for edge identification.

## Supplementary Information

Supplementary Information.
