# Supplementary material for: Identifying intracellular signaling modules and exploring pathways associated with breast cancer recurrence
Source: Sci Rep. 2021 Jan 11;11:385. doi: 10.1038/s41598-020-79603-5 (PMC7801429; doi:10.1038/s41598-020-79603-5)
Supplement: Supplementary file 1 — Supplementary Information. [file 41598_2020_79603_MOESM1_ESM.pdf]

Supplementary Material

Identifying intracellular signaling modules and exploring pathways associated with breast cancer recurrence

Xi Chen, Jinghua Gu, Andrew F. Neuwald, Leena Hilakivi-Clarke, Robert Clarke, Jianhua Xuan

Table S1. Candidate motifs for ER signaling, cell cycle, and apoptosis.

ER signaling		Cell cycle			Apoptosis	
V$AP1_C	V$NKX3A_01	V$ALPHACP1_01	V$E2F_Q6	V$OCT1_06	V$AHRARNT_01	V$E2F1_Q6_01
V$AP1_Q2	V$SP1_01	V$AP1_01	V$E2F_Q6_01	V$OCT1_07	V$AHRARNT_02	V$E47_01
V$AP1_Q2_01	V$SP1_Q2_01	V$AP1_C	V$E2F1_Q3	V$OCT1_B	V$AHRHIF_Q6	V$E47_02
V$AP1_Q4	V$SP1_Q4_01	V$AP1_Q2	V$E2F1_Q3_01	V$OCT1_Q5_01	V$AR_01	V$EBOX_Q6_01
V$AP1_Q4_01	V$SP1_Q6	V$AP1_Q2_01	V$E2F1_Q4	V$OCT1_Q6	V$AR_Q2	V$ER_Q6
V$AP1_Q6	V$SP1_Q6_01	V$AP1_Q4	V$E2F1_Q4_01	V$P53_01	V$AR_Q6	V$ER_Q6_02
V$AP1_Q6_01	V$SRF_01	V$AP1_Q4_01	V$E2F1_Q6	V$P53_02	V$ARNT_01	V$FOXO3_01
V$AP1FJ_Q2	V$SRF_C	V$AP1_Q6	V$E2F1_Q6_01	V$P53_DECAMER_Q2	V$ARNT_02	V$FOXO3A_Q1
V$CEBP_Q2_01	V$SRF_Q4	V$AP1_Q6_01	V$EBOX_Q6_01	V$SP1_01	V$ATF3_Q6	V$HAND1E47_01
V$CEBP_Q3	V$SRF_Q5_01	V$AP1FJ_Q2	V$ETS_Q4	V$SP1_Q2_01	V$CREB_01	V$MAX_01
V$CEBPB_01	V$SRF_Q5_02	V$ATF4_Q2	V$ETS_Q6	V$SP1_Q4_01	V$CREB_02	V$MYC_Q2
V$CEBPB_02	V$SRF_Q6	V$CETS1P54_01	V$ETS1_B	V$SP1_Q6	V$CREB_Q2	V$MYCMAX_01
V$CREB_01	V$STAT_01	V$CMYB_01	V$ETS2_B	V$SP1_Q6_01	V$CREB_Q2_01	V$MYCMAX_02
V$CREB_02	V$STAT_Q6	V$CREB_01	V$MYB_Q3	V$TAXCREB_01	V$CREB_Q3	V$MYCMAX_03
V$CREB_Q2	V$STAT1_01	V$CREB_02	V$MYB_Q5_01	V$TAXCREB_02	V$CREB_Q4	V$MYCMAX_B
V$CREB_Q2_01	V$STAT1_02	V$CREB_Q2	V$MYB_Q6	V$USF_01	V$CREB_Q4_01	V$MYOD_Q6_01
V$CREB_Q3	V$STAT1_03	V$CREB_Q2_01	V$MYOGNF1_01	V$USF_02	V$CREBATF_Q6	V$P53_01
V$CREB_Q4	V$STAT3_01	V$CREB_Q3	V$NF1_Q6	V$USF_C	V$DR3_Q4	V$P53_02
V$CREB_Q4_01	V$STAT3_02	V$CREB_Q4	V$NF1_Q6_01	V$USF_Q6	V$E12_Q6	V$P53_DECAMER_Q2
V$CREBATF_Q6	V$STAT4_01	V$CREB_Q4_01	V$NFY_01	V$USF_Q6_01	V$E2A_Q2	V$PBX_Q3
V$CREBP1_01	V$STAT5A_01	V$CREBATF_Q6	V$NFY_C	V$USF2_Q6	V$E2A_Q6	V$PBX1_01
V$CREBP1_Q2	V$STAT5A_02	V$CREBP1CJUN_01	V$NFY_Q6	V$YY1_01	V$E2F_02	V$PBX1_02
V$CREBP1CJUN_01	V$STAT5A_03	V$E2F_01	V$NFY_Q6_01	V$YY1_02	V$E2F_03	V$PPARA_02
V$ELK1_01	V$STAT5A_04	V$E2F_02	V$OCT_C	V$YY1_Q6	V$E2F_Q2	V$T3R_Q6
V$ELK1_02	V$STAT5B_01	V$E2F_03	V$OCT_Q6	V$YY1_Q6_02	V$E2F_Q3_01	V$TAL1_Q6
V$ETS_Q4	V$STAT6_01	V$E2F_Q2	V$OCT1_01		V$E2F_Q4_01	V$TAL1ALPHAE47_01
V$ETS_Q6	V$STAT6_02	V$E2F_Q3	V$OCT1_02		V$E2F_Q6_01	V$TAL1BETAE47_01
V$NFKB_C	V$TAXCREB_01	V$E2F_Q3_01	V$OCT1_03		V$E2F1_Q3	V$TAXCREB_01
V$NFKB_Q6	V$TAXCREB_02	V$E2F_Q4	V$OCT1_04		V$E2F1_Q3_01	V$TAXCREB_02
V$NFKB_Q6_01		V$E2F_Q4_01	V$OCT1_05		V$E2F1_Q6	V$WT1_Q6

Table S2. GibbsOS identified transcription factors that are up-regulated (+) or down-regulated (-) in the early-recurrence group of Loi data.

ER-signaling		Apoptosis	
V$STAT_01	+	V$FOXO3_01	-
V$STAT3_02	+	V$CREB_02	-
V$CREB_Q3	+	V$TAXCREB_01	-
V$STAT5A_02	+	V$AR_Q6	-
V$STAT5A_04	+	V$EBOX_Q6_01	-
V$CREB_Q4_01	+	V$CREB_Q2	-
V$TAXCREB_02	+	V$DR3_Q4	-
V$ELK1_02	+	V$WT1_Q6	-
V$CREB_Q4	+	V$CREB_Q4	-
V$CREBP1_01	+	V$TAL1ALPHAE47_01	-
V$ETS_Q6	+	V$PPARA_02	-
V$ELK1_01	+	V$E2A_Q6	-
V$CREB_Q2	+	V$HAND1E47_01	-
V$CEBPB_01	-	V$AHRARNT_02	-
V$CEBP_Q2_01	-	V$TAL1_Q6	-
V$CREB_02	-	V$TAXCREB_02	-
V$TAXCREB_01	-	V$CREB_Q4_01	-
V$ETS_Q6	-	V$ER_Q6	-
V$STAT1_03	-	V$E2F_Q2	-
V$STAT5A_02	-	V$CREB_01	-
V$CREB_Q4	-	V$CREB_Q2_01	-
V$AP1_Q6	-		
V$ELK1_02	-		
V$CREBP1_Q2	-		
V$CREB_01	-		
V$STAT_Q6	-		
V$SP1_Q4_01	-		
V$CREB_Q2	-		

Table S3. IMPALA identified pathway module genes from the Loi data.

Module 1	Module 2	Module 3	Module 4
BCR	CAV1	BRCA1	BRCA1
CREBBP	CSF1R	BRCA2	CCNA2
CSNK2A1	ERBB2	CCNA2	CDC2
EGR1	ESR1	CCNB1	CDC25C
ESR1	FYN	CDC2	CHUK
FOS	HCK	CDC25A	CSNK2A1
HDAC2	INPP5D	CDC25C	E2F1
HSP90AA1	JAK1	CHEK1	FAS
HSPA1A	LYN	E2F1	FYN
IGF1R	PECAM1	PBK	HSP90AA1
IRS1	PTPRC	TGFB1	LCK
IRS2	STAT3	TGFBR2	PRKCA
JUN	STAT5A	TOP2A	TNFRSF1A
PTPN11	WAS	TP53	WAS
RPS6KA1			YWHAQ
SRC			
STAT3			
STMN1			
TOP2A			
TSC2			
YWHAQ			
YWHAZ			

Table S4. GibbsOS identified transcription factors that are up-regulated (+) or down-regulated (-) in the early-recurrence group of Symmans data.

ER-signaling		Cell cycle		Apoptosis	
V$SP1_Q4_01	+	V$CREB_Q4_01	+	V$TAL1BETAE47_01	+
V$AP1_C	+	V$NFY_Q6	+	V$CREB_Q4_01	+
V$AP1_Q6_01	+	V$AP1_Q4	+	V$P53_DECAMER_Q2	+
V$AP1_Q4_01	+	V$USF2_Q6	+	V$E2F_Q2	+
V$STAT1_02	+	V$AP1_Q4_01	+	V$CREBATF_Q6	+
V$AP1_Q2	+	V$USF_Q6_01	+	V$MYOD_Q6_01	+
V$CREB_Q3	+	V$AP1_Q6	+	V$E2A_Q6	+
V$AP1FJ_Q2	+	V$CMYB_01	+	V$MYCMAX_B	+
V$AP1_Q6	+	V$CETS1P54_01	+	V$TAL1_Q6	+
V$STAT3_02	+	V$NFY_Q6_01	+	V$CREB_02	+
V$CREB_Q2_01	+	V$NF1_Q6	+	V$E2A_Q6	-
V$STAT1_02	-	V$SP1_Q4_01	+	V$CREB_Q4_01	-
V$SP1_01	-	V$E2F_Q4	+	V$TAL1BETAE47_01	-
V$SP1_Q6	-	V$USF_C	+	V$EBOX_Q6_01	-
V$CREB_Q2_01	-	V$E2F_Q6	+	V$ARNT_02	-
V$CREB_02	-	V$CREB_Q2_01	+	V$PBX1_01	-
V$CEBP_Q3	-	V$YY1_Q6_02	+	V$CREB_Q4	-
		V$NFY_01	+	V$CREB_01	-
		V$MYB_Q5_01	+		
		V$CREB_Q3	+		
		V$AP1_Q6_01	+		
		V$CREBATF_Q6	+		
		V$OCT1_Q5_01	-		
		V$MYB_Q6	-		
		V$SP1_Q6	-		
		V$YY1_Q6_02	-		
		V$MYB_Q5_01	-		
		V$USF_Q6_01	-		
		V$CREB_Q2_01	-		
		V$ETS1_B	-		
		V$ETS2_B	-		
		V$YY1_02	-		
		V$USF_02	-		
		V$NFY_C	-		
		V$OCT1_B	-		

Table S5. IMPALA identified pathway module genes from the Symmans data.

Module 1	Module 2	Module 3	Module 4
BRCA1	ESR1	CDC2	CDK2
CDC2	HCK	E2F1	EGFR
CDC25C	INPP5D	EGFR	ETS1
CHUK	JAK1	FAS	FAS
CSNK2A1	JUN	GRB2	FOS
E2F1	KHDRBS1	HCK	HCK
GRB2	LCK	INPP5D	INPP5D
HDAC2	LCP2	INSR	JAK1
HSP90AA1	LYN	LCP2	JUN
IGF1R	MAP4K1	LYN	LCK
INSR	PIK3R1	MAP4K1	LCP2
MAPK1	PTPN6	MET	LYN
PTPN11	SHC1	PTPN6	MAPK1
SRC	SOS1	TNFRSF1A	MYOD1
STMN1	STAT3		NR3C1
TNFRSF1A			SP1
TOP2A			STAT3
YWHAQ			

Table S6. Average precision of IMPALA and competing algorithms under different settings.

**Average precision**	Noise Level	Type I structure		Type II structure	
		gene	edge	gene	edge
**IMPALA**	0.2	**0.960**	**0.942**	**0.900**	**0.836**
Random Color Coding		0.806	0.701	0.851	0.669
Edge Orientation		0.807	0.695	0.777	0.473
ILP		0.406	N/A	0.586	N/A
**IMPALA**	0.5	**0.910**	**0.835**	**0.806**	**0.690**
Random Color Coding		0.801	0.592	0.768	0.568
Edge Orientation		0.780	0.569	0.738	0.441
ILP		0.387	N/A	0.570	N/A
**IMPALA**	0.8	**0.843**	**0.700**	**0.800**	**0.466**
Random Color Coding		0.755	0.481	0.715	0.417
Edge Orientation		0.716	0.472	0.677	0.351
ILP		0.364	N/A	0.485	N/A

Table S7. Average precision for pathway identification under different proportion of false connections (PFC) in simulated pathways.

**Average precision**	PFC	Type I structure		Type II structure	
		gene	edge	gene	Edge
**IMPALA**	10%	**0.835**	**0.635**	**0.768**	**0.660**
Random Color Coding		0.695	0.426	0.716	0.517
Edge Orientation		0.675	0.417	0.737	0.490
ILP		0.372	N/A	0.517	N/A
**IMPALA**	25%	**0.634**	**0.309**	**0.770**	**0.542**
Random Color Coding		0.439	0.132	0.761	0.435
Edge Orientation		0.418	0.185	0.701	0.382
ILP		0.348	N/A	0.472	N/A
**IMPALA**	50%	**0.494**	**0.183**	**0.682**	**0.525**
Random Color Coding		0.424	0.116	0.664	0.410
Edge Orientation		0.381	0.136	0.587	0.325
ILP		0.327	N/A	0.473	N/A

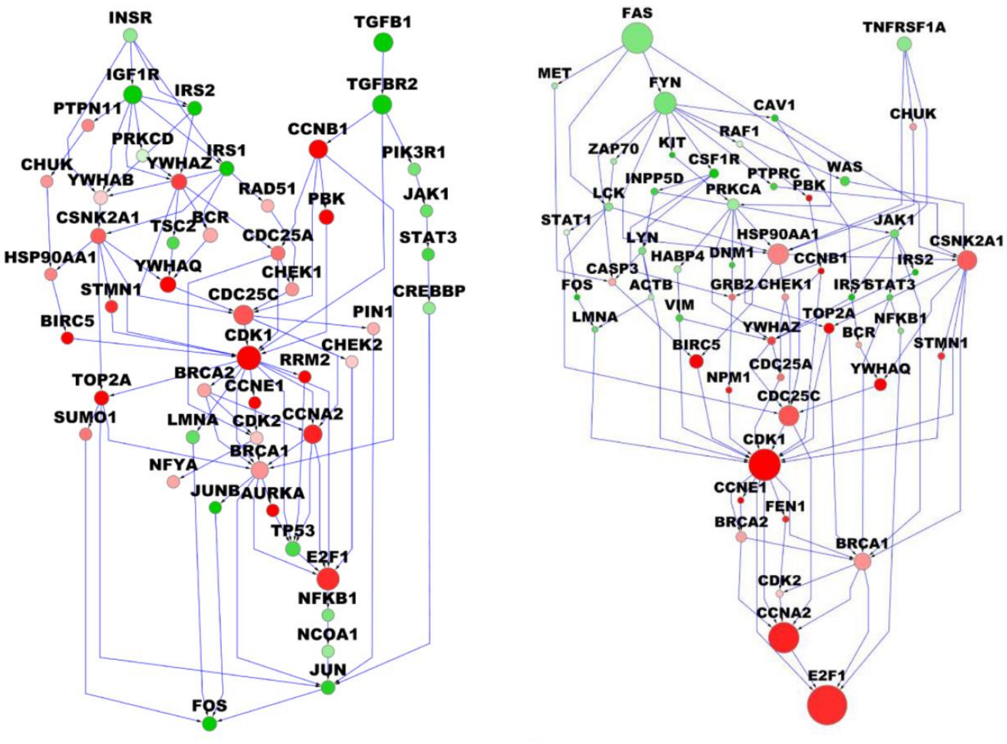

Cell cycle Apoptosis

Figure S1. Cell cycle and apoptosis signaling pathway networks identified by IMPALA using Loi data. Gene colors represent the log2 fold change of gene expression between ‘early recurrence’ and ‘late recurrence’ patients in the Loi dataset (red: over-expressed in ‘early recurrence’ group; green: over-expressed in ‘late recurrence’ group). Gene size is proportional to the probability (sampling frequency) estimated by GIST.

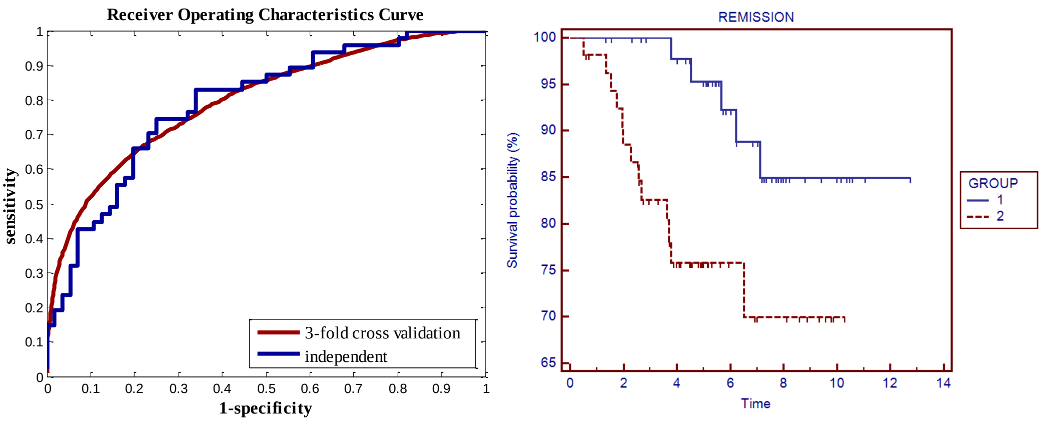

(a) (b)

Figure S2. Prediction analysis of ER signaling modules identified from Loi data. (a) Threefold cross-validation using Loi data returned area of ROC curve (AUC) 0.8. Independent test of the classifier on Symmans data returned AUC of 0.79. (b) Kaplan Meier plot of predicted grouping of Symmans samples (group 1 for 'late recurrence' and group 2 for 'early recurrence') returned a hazard ratio of 3.26 (p-value = 1.6e-2).

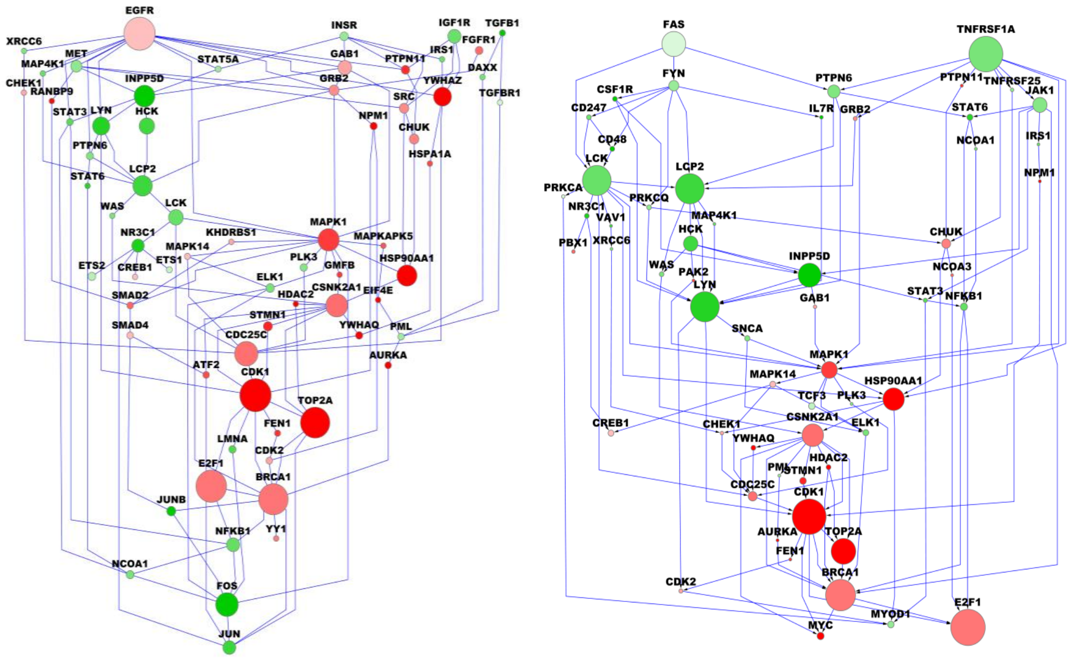

Cell cycle Apoptosis

Figure S3. Cell cycle and apoptosis signaling pathway networks identified by IMPALA using Symmans data. Gene colors represent the log2 fold change of gene expression between 'early recurrence' and 'late recurrence' patients in the Symmans dataset (red: over-expressed in 'early recurrence' group; green: over-expressed in 'late recurrence' group). Gene size is proportional to the probability (sampling frequency) estimated by GIST.

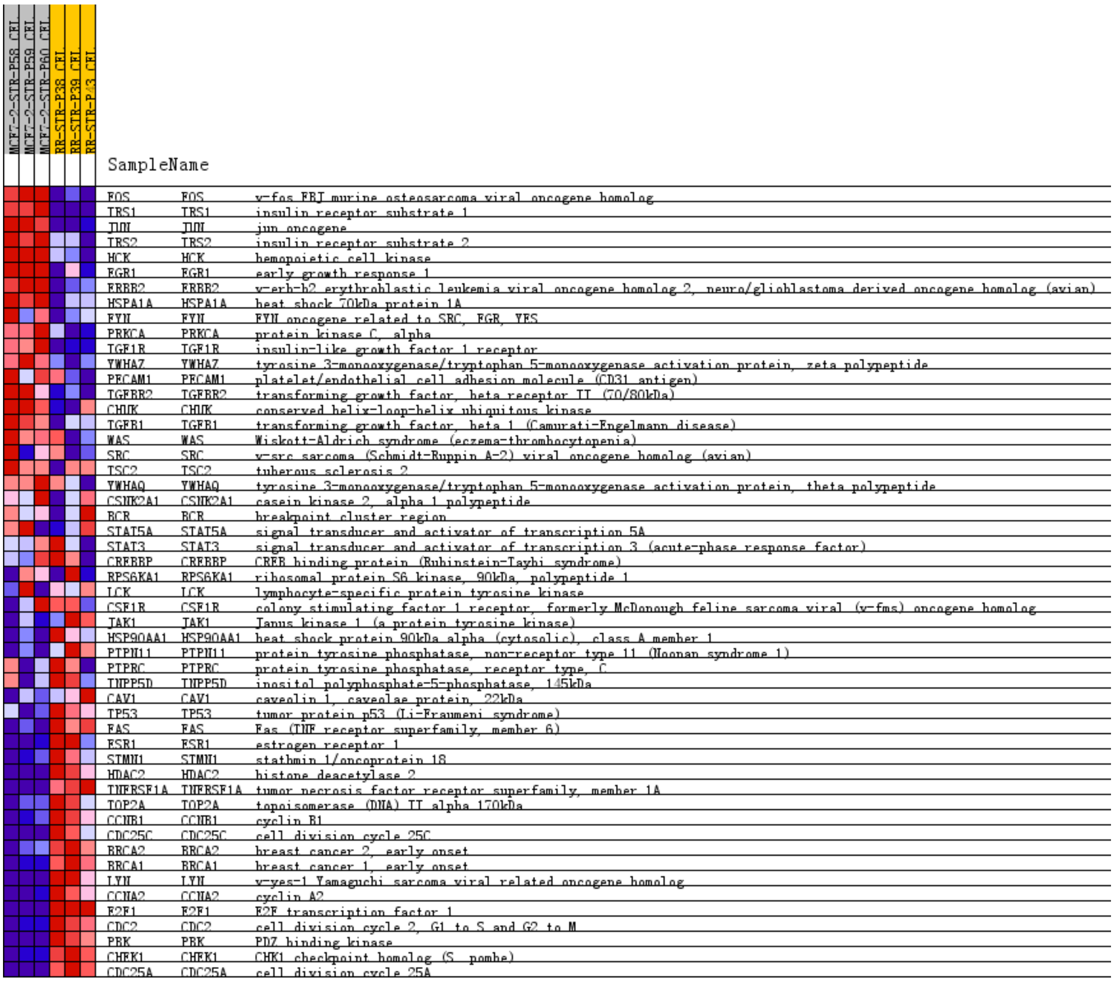

Figure S4. Gene expression in MCF7-STR and MCF7R-STR cell lines for IMPALA identified genes using Loi data.

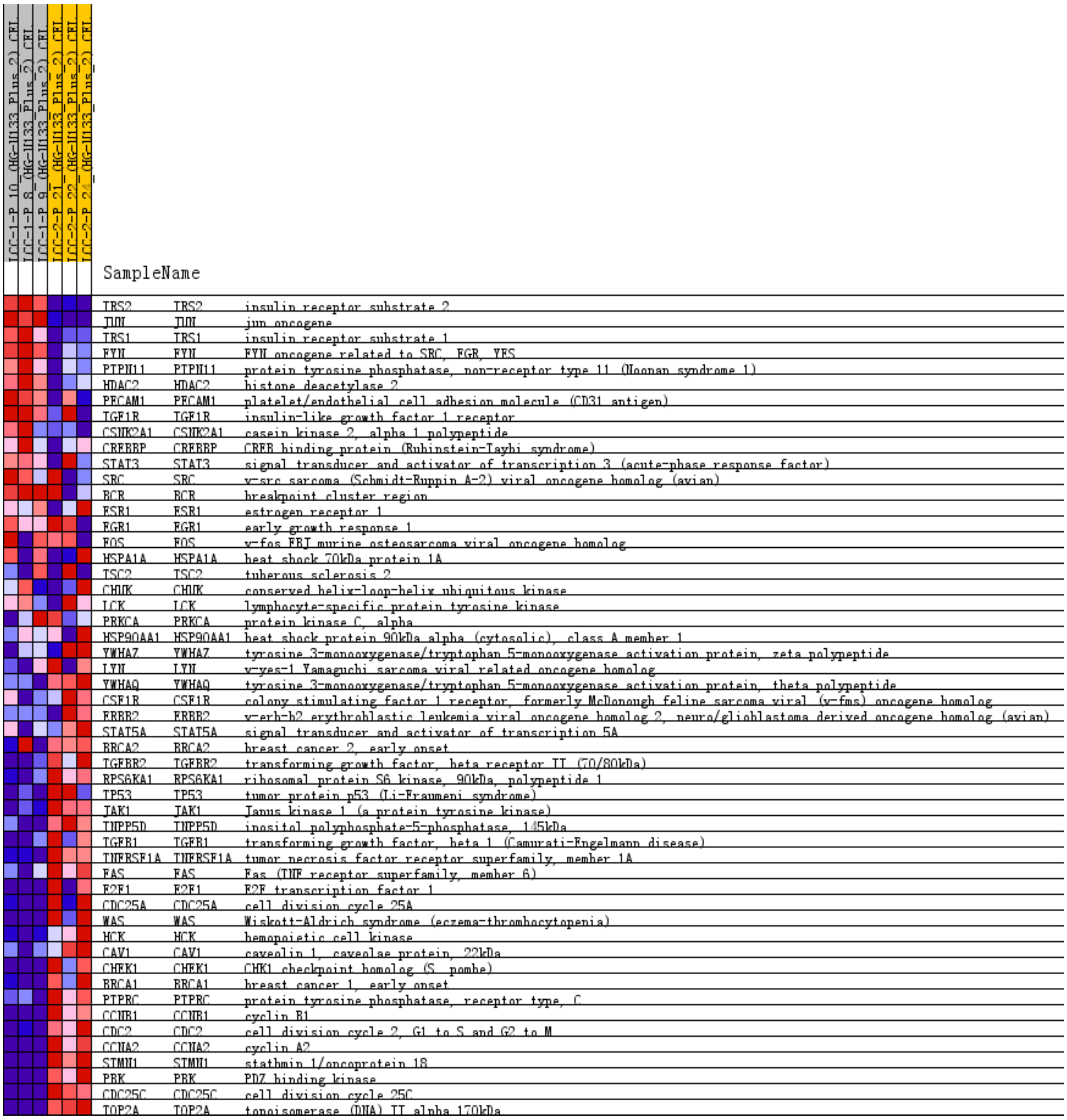

Figure S5. Gene expression in LCC1 and LCC2 cell lines for IMPALA identified genes using Loi data.

Supplementary Methods

Building flow network

GIST applies Gibbs sampling to a regularized structure which we refer to as "flow network". Given the source and target gene(s), we build a directed pathway flow network of L layers from the original PPI as shown in **Fig. S6**. First, we start from the source genes (genes in the first layer) and search their neighbors in the PPI network. The direct neighbors of the source genes are included into the second layer, based on which we successively define the third layer, forth layer, etc. This is called the "forward search" of the PPI network, and the target gene will present at the Lth layer (the target gene can also show up in the upper layers if there is a path between source and target that has length smaller than L). In the meanwhile, we also perform a "backward search" starting from the target gene and rebuild L layers in the reverse direction. For each layer we only keep the genes that present at both "forward" and "backward" networks and obtain the final flow network.

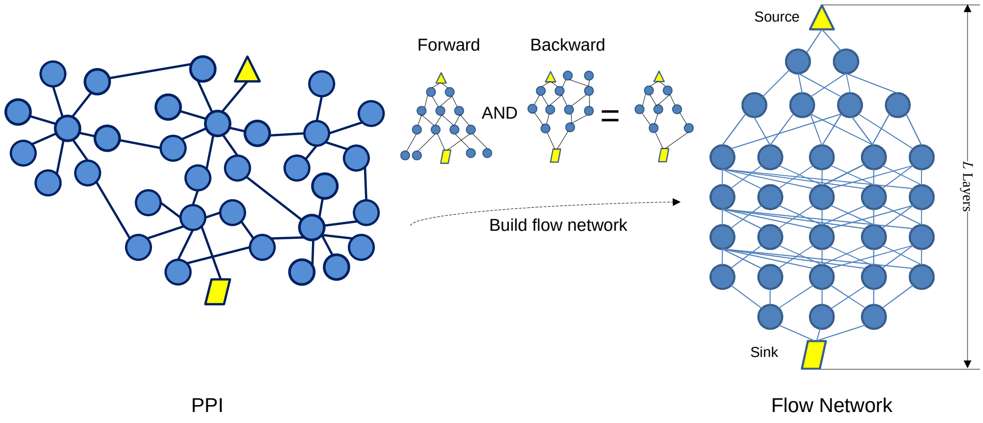

Figure S6. An illustration of constructing flow network from PPI data

Sampling flow network with a modified Gibbs sampler and Markov property

We illustrate the sampling procedure as shown in **Fig. S7**. The current pathway is highlighted in the shaded area. A Gibbs sampler updates one gene θ_i at a time, and iteratively updates the other module members. Suppose we want to update the third gene θ_3 in the pathway. Based on the flow network, three genes in the third layer (marked 1, 2, and 3) are potential candidates that connect the existing genes to the second and fourth layer. We calculate the pathway probabilities for all three corresponding pathways and then probabilistically accept one of the genes to update the previous gene (gene 2 is selected to update θ_3). We also correspondingly update the edges of the new gene. This procedure will be sequentially applied to the fourth, fifth until the Lth layer, and will be repeated for many iterations until convergence.

In order that through enough sampling iterations, the estimated distribution will be a stationary distribution that is irrelevant to its initial states, the proposed Gibbs sampler should have some basic properties such as irreducibility and ergodicity. Unfortunately, this property cannot always be satisfied when we sample pathways from the PPI network and only allows changing one layer in the current path at a time. Here we propose a simple modification of the previous Gibbs sampler by introducing a small baseline sampling frequency δ to states (pathway configurations) that are not allowed by the initial flow network, so that any two states in $\mathbf{\Theta}$ communicate with each other. Edges connecting genes in two adjacent layers that are not connected in the original PPI are referred to as "pseudo-edges" (non -directed lines in **Fig. 8**). Then, genes in any two adjacent layers are mutually connected regardless of whether they are truly connected in the original PPI network or not. The Gibbs sampling process on the modified flow network has defined an irreducible Markov chain that draw samples (states) from an enlarged state space $\mathbf{\Theta}'$ (Fig. S8(b)) and $\mathbf{\Theta} \subset \mathbf{\Theta}'$, where $\mathbf{\Theta}$ is the state space of all "valid pathways" defined by the original flow network (Fig. S8(a)).

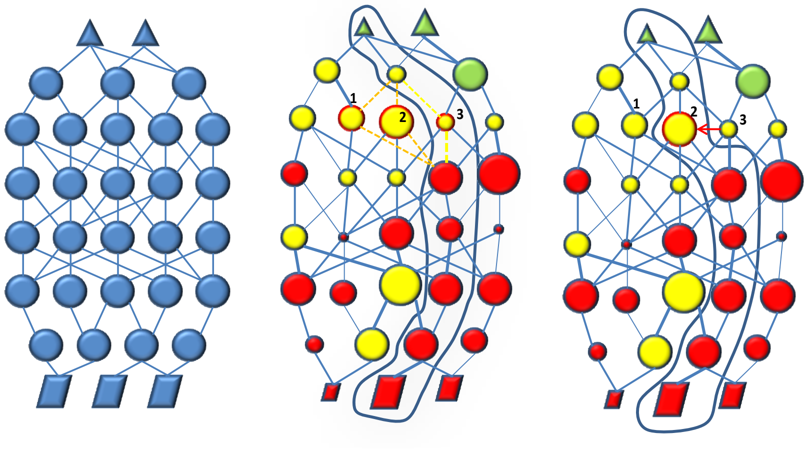

(a) (b) (c)

Figure S7. Sampling on the flow network. (a) A flow network was constructed among given source(s) and target(s) using protein-protein interactions where 'triangle' represented source genes; 'cycle' represented pathway genes; 'rectangular' represented target transcription factors. (b) Genes and edges were assigned weights based on potential functions as defined in the main text, using gene expression data. Different gene colors represented different subcellular compartments ('green' for membrane receptors; 'yellow' for cytoplasm genes; 'red' for nucleus transcription factors. (c) A sampled pathway by GIST from the flow network.

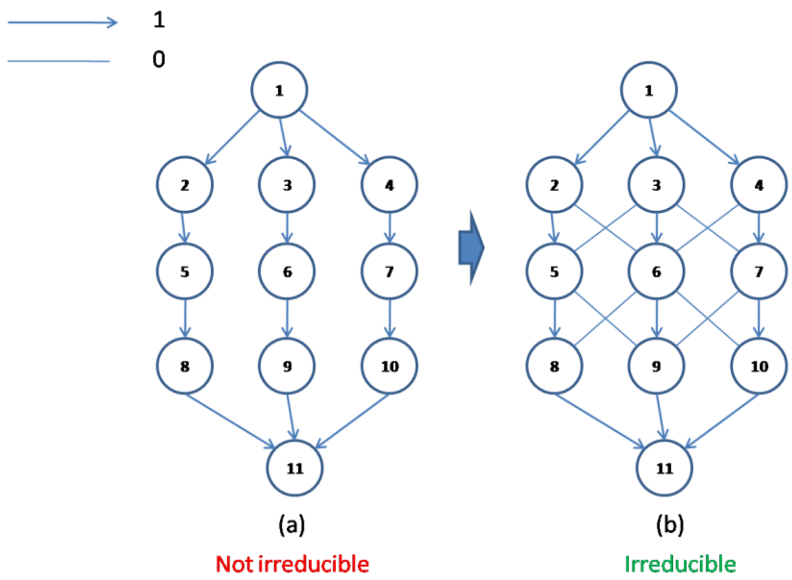

Figure S8. An example of irreducibility for pathway sampling

Truncation of pathways samples to estimate edge probability

When the number of genes in Ω is large, the sample space $\mathbf{\Theta}$ becomes huge. In this case, it is not computationally feasible to sample all possible pathways to calculate the posterior probability of every directed edges. Practically, we are interested in the top ranked pathway samples with the highest inter-correlation. Therefore, we offer a non-normalized edge probability based on truncated samples as follows:

$$p_{i,j}^{K} = \sum_{\boldsymbol{\theta} \in \mathbf{\Theta}^{K}} \left(V_1(\boldsymbol{\theta}) + V_2(\boldsymbol{\theta}) \right) \cdot P\left(e_{i,j} = 1 \middle| \boldsymbol{\theta} \right)$$
$$\sim \frac{\sum_{\boldsymbol{\theta} \in \mathbf{\Theta}^{K}} \left(V_1(\boldsymbol{\theta}) + V_2(\boldsymbol{\theta}) \right) \cdot P\left(e_{i,j} = 1 \middle| \boldsymbol{\theta} \right)}{\sum_{\boldsymbol{\theta} \in \mathbf{\Theta}^{K}} \left(V_1(\boldsymbol{\theta}) + V_2(\boldsymbol{\theta}) \right)} = P^{K}(e_{i,j} = 1), \qquad \text{(S1)}$$

where $\mathbf{\Theta}^{K}$ denotes top K truncated pathway samples. $p_{i,j}^{K}$ is not a probability but a score function for ranking valid pathway samples. In Eq. (S1) we only used gene and edge potentials because samples from $\mathbf{\Theta}^{K}$ have already been well constrained by prior knowledge (e.g., cellular locations). The confidence of edge direction was defined as follows:

$$q^{K}\left(e_{i,j}\right) = \frac{p_{i,j}^{K}}{p_{i,j}^{K} + p_{j,i}^{K}} = \frac{P^{K}(e_{i,j} = 1)}{P^{K}(e_{i,j} = 1) + P^{K}(e_{j,i} = 1)}, \text{ s.t. } \max\left(p_{i,j}^{K}, p_{j,i}^{K}\right) \neq 0 \,. \qquad \text{(S2)}$$

Simulating alternative and crosstalk pathways

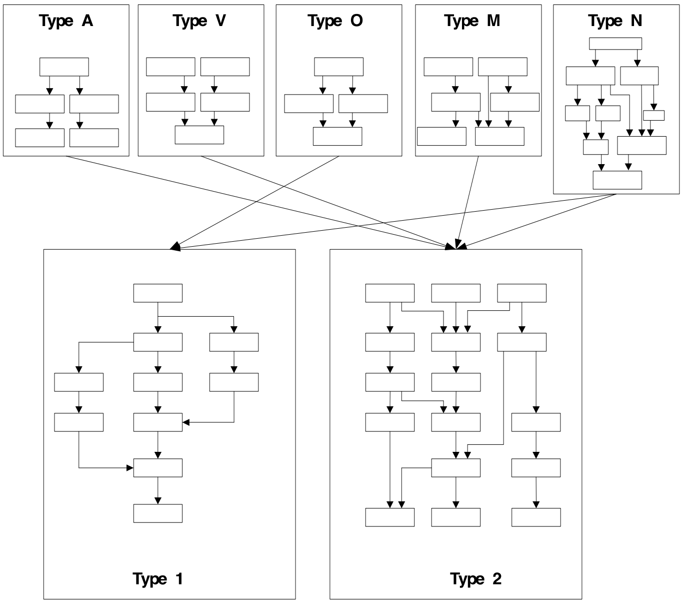

Figure S9. Type I and Type II alternative pathway structures.

To construct alternative pathways, Gong *et al.* proposed five basic types of alternative pathways including: A (divergent), V (convergent), O (single/multiple), M (multiple/multiple) and N (nested) [1]. We summarize these into two major types of alternative pathway structures: type I, alternative pathways between single source and single target; type II, alternative pathways among multiple sources and multiple targets. A schematic diagram of the two pathway structures is shown in **Fig. S9**. It can be found that type I structure is a special case of type N (nested) pathway between a single source gene and a single target gene, which embraces type O (single/multiple) pathways as sub-components. Type II structure is actually a more general case of the type N (nested) structure among multiple source genes and multiple target genes. It has a mixture structure of type A (divergent), type V (convergent) and type M (multiple/multiple) pathways.

Both type I and type II structures are designed to study alternative signal transduction, while the latter is also used to model crosstalk among multiple pathways. To generate simulation pathways for each structure, a subnetwork centered at some putative hub genes within the human PPI network was selected as the base topology. Genes that are involved in canonical pathways (MAPK, ERBB, JAK/STAT, et al.) were also extracted from the knowledge database [2,3] as the candidate pool for building ground truth pathways. We also collected subcellular location information for the human proteome. To keep the models simple and logical for this study, we assumed that a valid path

should start from the extracellular space/plasma membrane and end in the nucleus. A mixture model was used to synthesize the edge z-scores plus different levels of noise so that we can simulate real biological scenarios with experimental/biological noise. An exhaustive search was conducted to obtain the ground truth distributions of both genes and edges.

Gene expression noise level = 0.2

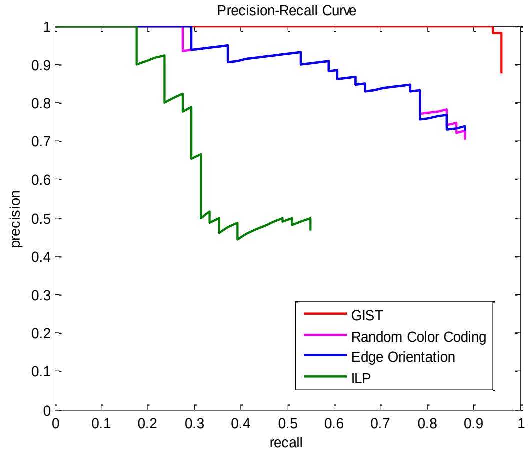

Pathway gene identification

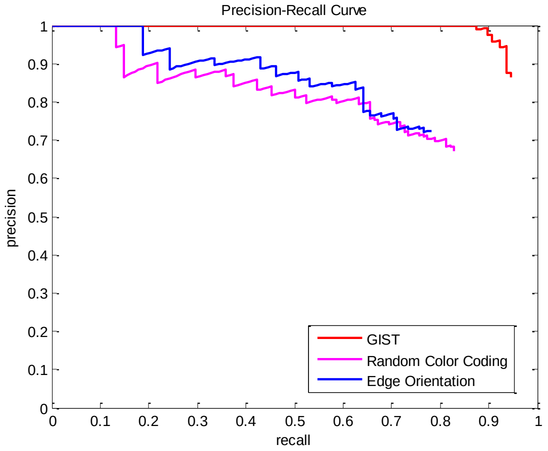

Pathway interaction identification

Gene expression noise level = 0.5

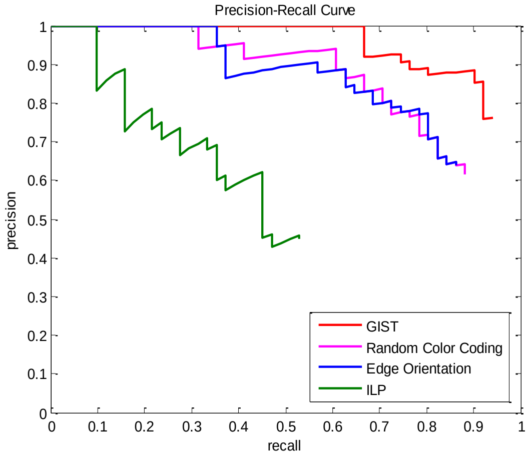

Pathway gene identification

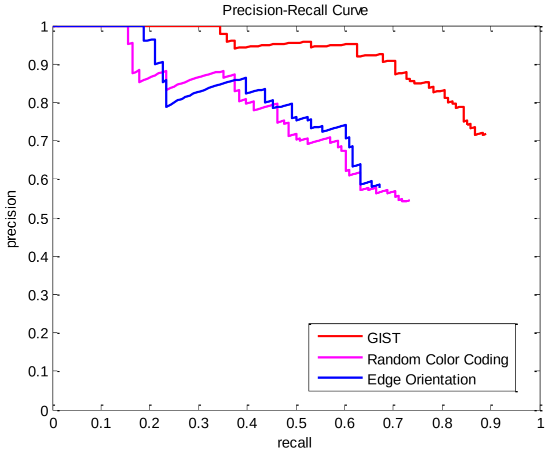

Pathway interaction identification

Gene expression noise level = 0.8

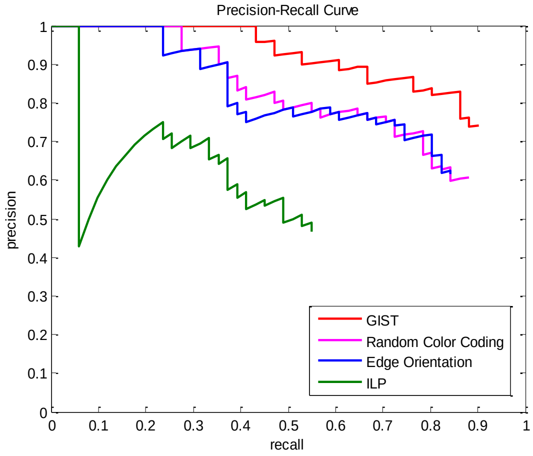

Pathway gene identification

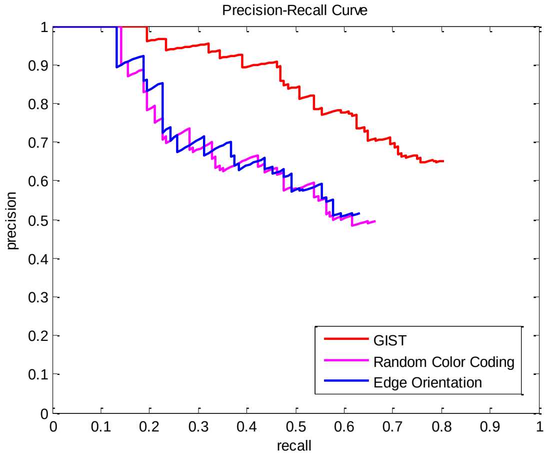

Pathway interaction identification

Figure S10. Precision-Recall curves for pathway gene/interaction identification on type I pathway structure under different noise levels.

Gene expression noise level = 0.2

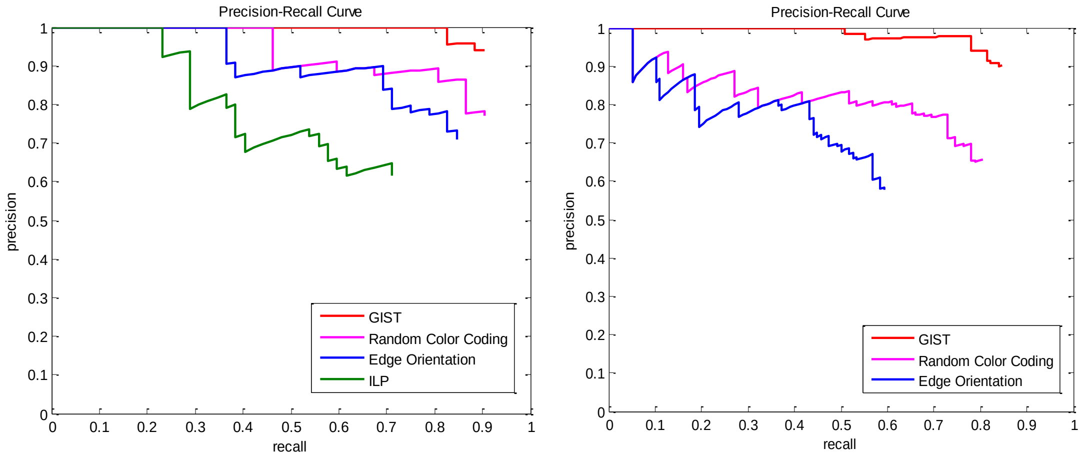

Pathway gene identification

Pathway interaction identification

Gene expression noise level = 0.5

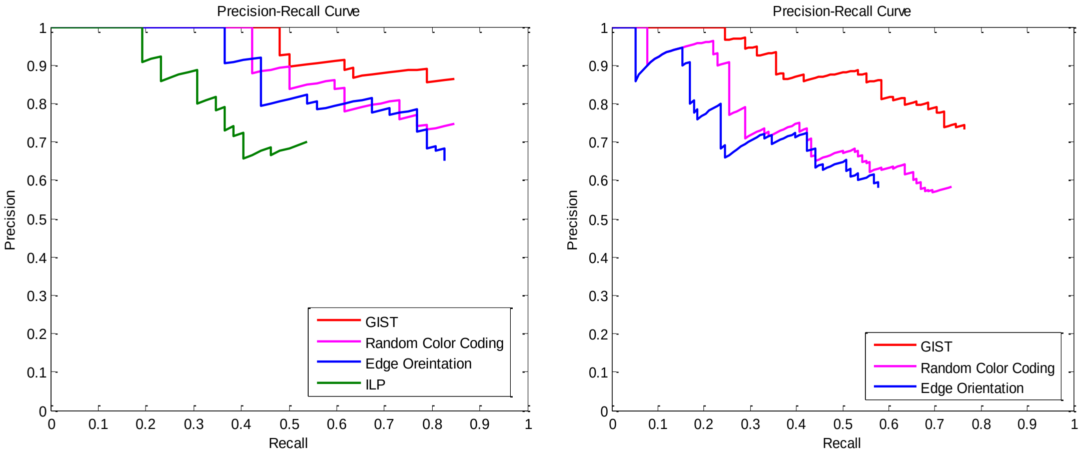

Pathway gene identification

Pathway interaction identification

Gene expression noise level = 0.8

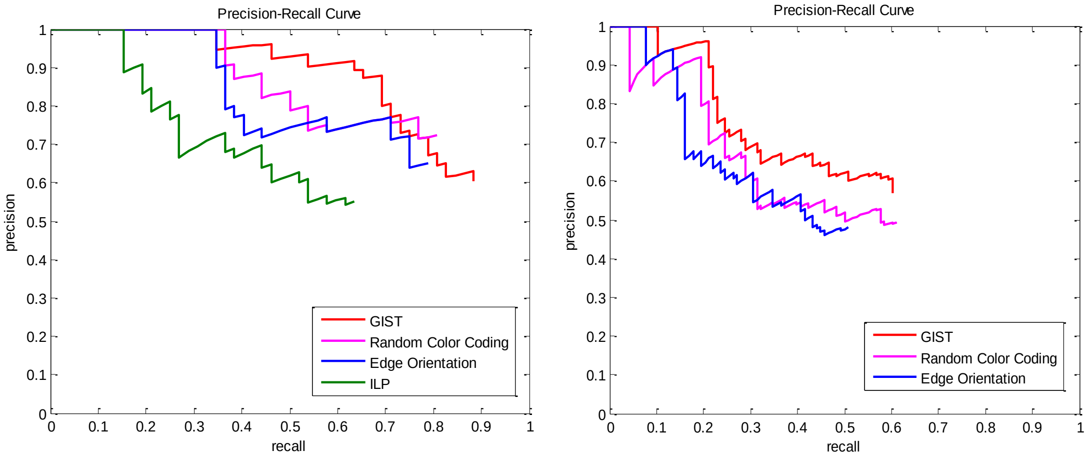

Pathway gene identification

Pathway interaction identification

Figure S11. Precision-Recall curves for pathway gene/interaction identification on type II pathway structure under different noise levels.

Pathway network false interaction rate = 10%

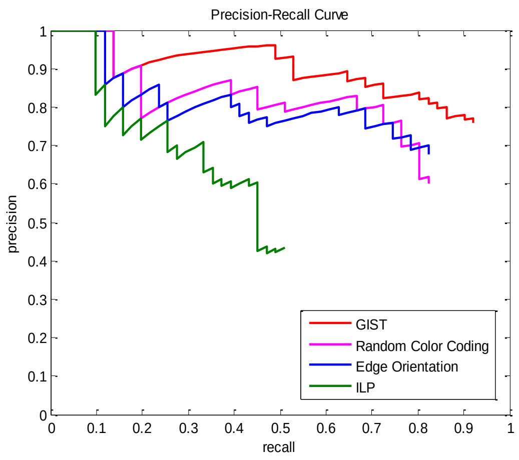

Pathway gene identification

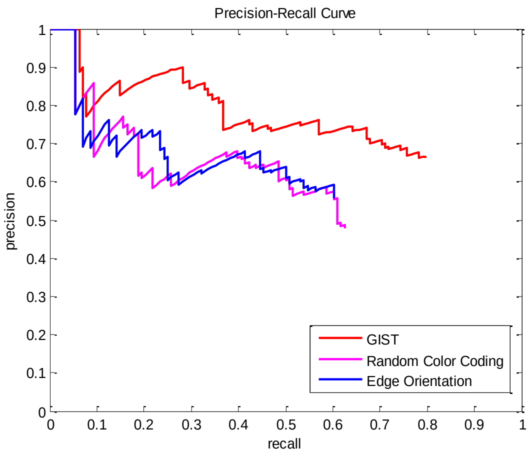

Pathway interaction identification

Pathway network false interaction rate = 25%

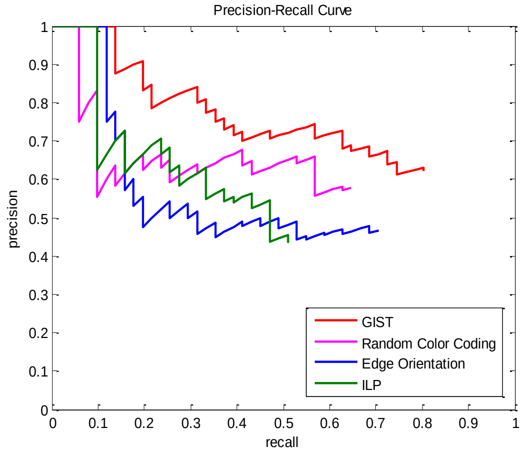

Pathway gene identification

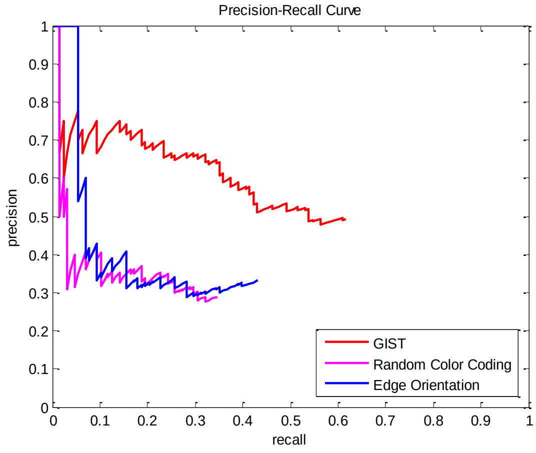

Pathway interaction identification

Pathway network false interaction rate = 50%

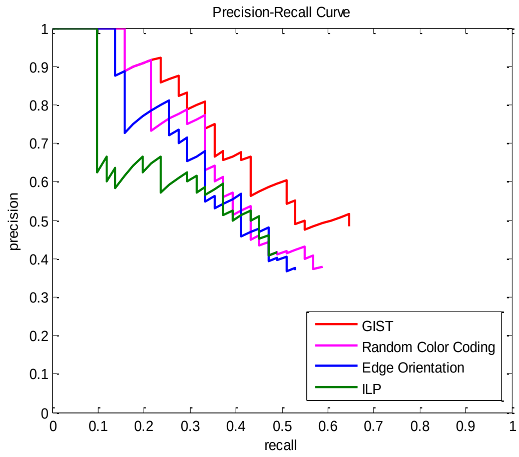

Pathway gene identification

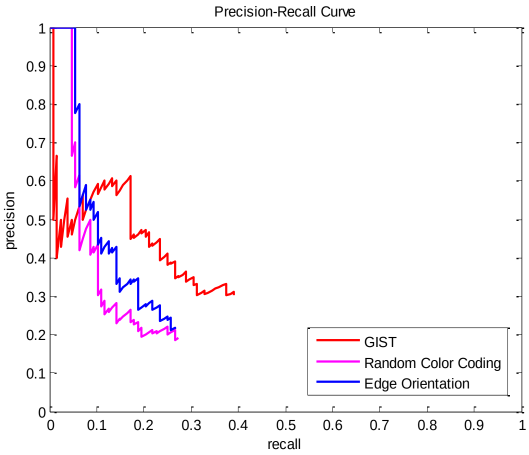

Pathway interaction identification

Figure S12. Precision-Recall curve for gene/edge identification on type I pathway structure under different false interaction rates in simulated pathways.

Pathway network false interaction rate = 10%

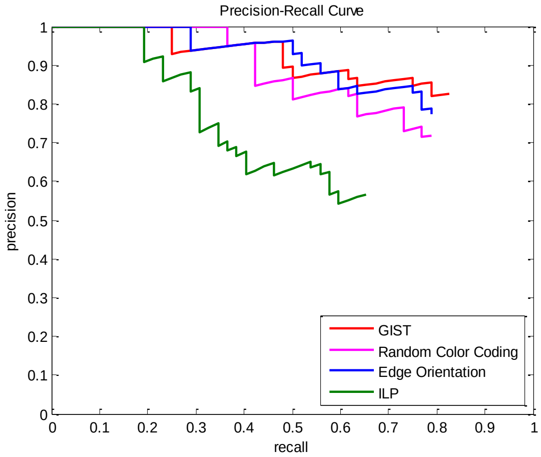

Pathway gene identification

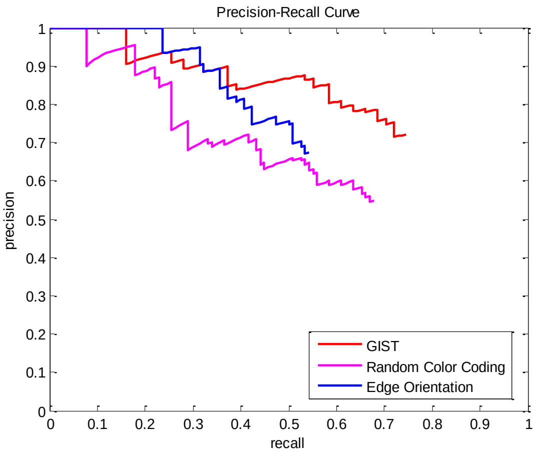

Pathway interaction identification

Pathway network false interaction rate = 25%

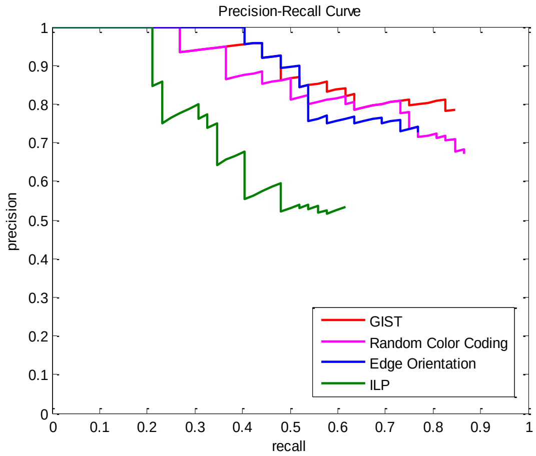

Pathway gene identification

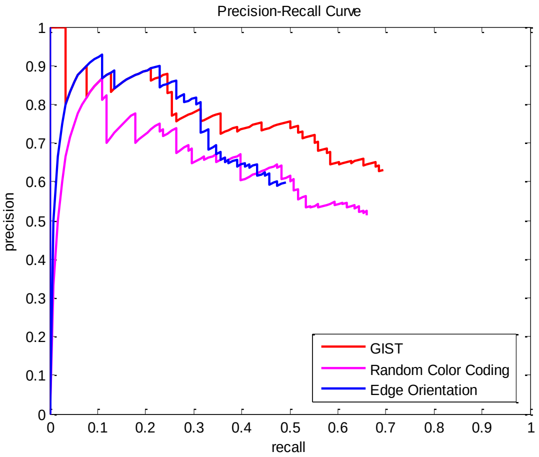

Pathway interaction identification

Pathway network false interaction rate = 50%

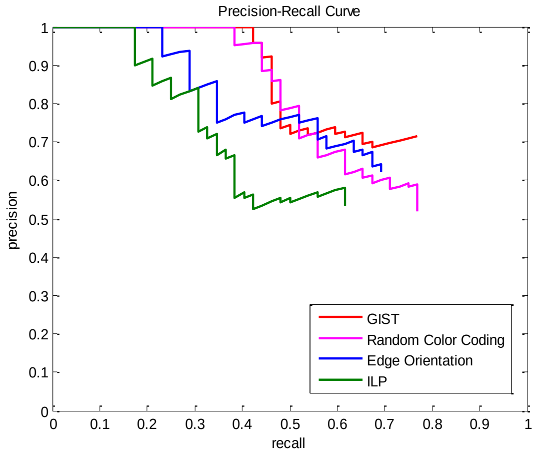

Pathway gene identification

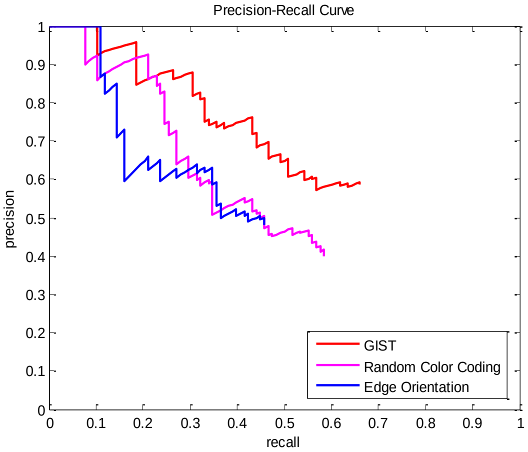

Pathway interaction identification

Figure S13. Precision-Recall curve for gene/edge identification on type II pathway structure under different false interaction rates in simulated pathways.

References

1 Gong, Y. & Zhang, Z. Alternative signaling pathways: when, where and why? *FEBS Lett* **579**, 5265-5274, (2005).

2 Dennis, G., Jr. *et al.* DAVID: Database for Annotation, Visualization, and Integrated Discovery. *Genome Biol* **4**, P3, (2003).

3 Kanehisa, M., Goto, S., Sato, Y., Furumichi, M. & Tanabe, M. KEGG for integration and interpretation of large-scale molecular data sets. *Nucleic Acids Res* **40**, D109-114, (2012).
